# CAPTYN, a Blood-Based Machine-Learning Model Predicting Clinical Benefit of Atezolizumab–Bevacizumab in Hepatocellular Carcinoma: Validation in the IMbrave150 Trial

**DOI:** 10.34133/cancomm.0059

**Published:** 2026-10-19

**Authors:** Gae Hoon Jo, Sohyun Hwang, Bernhard Scheiner, Seonjeong Woo, Won Suk Lee, Beodeul Kang, Jung Sun Kim, Ho Yeong Lim, Chansik An, Dong Yun Kim, Inyoung Kim, Dong-hyuk Heo, Matthias Pinter, Beom Kyung Kim, Chan Kim, Hong Jae Chon

**Affiliations:** ^1^ Department of Life Science, Graduate School, CHA University, Seongnam, Republic of Korea.; ^2^ Department of Pathology, CHA Bundang Medical Center, CHA University School of Medicine, Seongnam, Republic of Korea.; ^3^ Division of Gastroenterology and Hepatology, Department of Medicine III, Medical University of Vienna, Vienna, Austria.; ^4^ Vienna Liver Cancer Study Group, Division of Gastroenterology and Hepatology, Department of Medicine III, Medical University of Vienna, Vienna, Austria.; ^5^ Department of Medical Oncology, CHA Bundang Medical Center, CHA University School of Medicine, Seongnam, Republic of Korea.; ^6^ Department of Radiology, CHA Bundang Medical Center, CHA University School of Medicine, Seongnam, Republic of Korea.; ^7^ Department of Internal Medicine, Yonsei University College of Medicine, Seoul, Republic of Korea.; ^8^ Yonsei Liver Center, Severance Hospital, Seoul, Republic of Korea.; ^9^ Theragen Bio Co., Ltd., Seongnam, Republic of Korea.; ^10^ Department of Biochemistry and Molecular Biology, Graduate School of Medical Science, Brain Korea 21 Project, Yonsei University College of Medicine, Seoul, Republic of Korea.

Hepatocellular carcinoma (HCC) is the most common primary liver cancer. Without appropriate surveillance, most patients are diagnosed with advanced-stage disease requiring systemic therapy [1], for which atezolizumab plus bevacizumab (Ate/Bev) has replaced tyrosine kinase inhibitors (TKIs) as the preferred first-line standard [2], alongside emerging dual-immunotherapy regimens [1]. However, these treatments benefit only a subset of patients and responses vary across regimens, motivating the need for predictive tools to guide patient selection [3]. Currently, several prognostic models are used in advanced HCC, including the CRAFITY score (C-reactive protein [CRP] and α-fetoprotein [AFP]) [4], the ALBI grade (albumin and total bilirubin) [5], and the CRAPT-M model, which incorporates 5 clinical variables, including protein induced by vitamin K absence or antagonist-II (PIVKA-II), a biomarker not routinely measured [6]. However, none of these models jointly captures the 4 biological dimensions critical for predicting Ate/Bev efficacy: systemic inflammation, tumor burden, hepatic reserve, and immune status [7]. Here, we developed CAPTYN, a machine-learning model that uses 6 widely available blood biomarkers and was trained on a multinational Asian–European cohort. We then externally validated it in the phase III IMbrave150 trial and an independent real-world cohort and explored its treatment specificity in TKI-treated cohorts.

We screened 949 patients with HCC from 3 hospitals in Korea and Austria and the IMbrave150 trial. After exclusion, 737 eligible patients were analyzed across training (CHA Bundang Medical Center, Korea, and Medical University of Vienna, Austria; *n* = 354), external validation (IMbrave150, *n* = 99; Yonsei University Health System [YUHS], Korea, *n* = 184), and TKI-treated control (sorafenib [SOR], *n* = 28; lenvatinib [LEN], *n* = 72) cohorts (Fig. S1). Baseline characteristics are summarized by cohort in Table S1. Median follow-up was 24.7 (95% confidence interval [CI], 23.4 to 25.5) months. Clinical benefit, the primary outcome, was defined as complete response, partial response, or stable disease with progression-free survival (PFS) ≥ 6 months per Response Evaluation Criteria in Solid Tumors v1.1; all others were classified as nonclinical benefit. Clinical outcomes by cohort are summarized in Table S2.

Detailed methods, including the feature-selection process (Fig. S2), are provided in the Supplementary Methods. From 18 baseline clinical variables, we excluded PIVKA-II (not routinely available), the subjective scores (Child–Pugh class and Eastern Cooperative Oncology Group performance status [ECOG PS]), and prothrombin time–international normalized ratio (PT-INR; redundant with albumin and total bilirubin; Fig. S3), leaving 14 variables. Univariate Cox regression for overall survival (OS) and PFS identified 9 candidate predictors (Table S3). Extreme gradient boosting (XGBoost) was selected among 4 algorithms based on the 5-fold cross-validated area under the curve (AUC) for nonclinical benefit classification (Fig. S4), and recursive feature elimination yielded 6 final predictors: CRP, AFP, platelet count, total bilirubin, lymphocyte count, and neutrophil count (Fig. S5). After hyperparameter optimization, the final XGBoost classifier achieved an internal 5-fold cross-validated AUC of 0.68 (Fig. S6); the apparent training-set AUC was 0.93 (Fig. S7). The model outputs a continuous probability of nonclinical benefit, with 0.486 as a Youden-derived reference cutoff. In a worst-case sensitivity analysis imputing 25 patients with missing responses as nonclinical benefit (Fig. S1), overall performance was unchanged (Table S4).

The model was named CAPTYN based on the initial letters of its predictors ranked by feature importance (Fig. S8), with “Y” representing lymphocytes. Elevated CRP, AFP, and total bilirubin and reduced lymphocyte counts favored nonclinical benefit, whereas platelet and neutrophil counts exhibited asymmetric U-shaped effects with both extremes associated with nonclinical benefit (Fig. S9). CAPTYN integrates 4 complementary clinical–biological dimensions relevant to Ate/Bev: systemic inflammation, represented by CRP, which may reflect host- and tumor-associated immunosuppressive signals that attenuate antitumor immunity; tumor burden, represented by AFP, which is associated with an aggressive HCC phenotype and may capture disease activity beyond radiologic imaging; hepatic reserve, represented by total bilirubin, which influences treatment tolerance; and peripheral immune status, represented by lymphocyte and neutrophil counts, which approximate the balance between adaptive immunity and innate/myeloid-driven immunity. Platelet count serves as a cross-domain marker linking inflammation, tumor burden, and hepatic reserve. Altogether, these dimensions align with the molecular correlates of Ate/Bev response identified in the GO30140 and IMbrave150 trials [8], reflecting the dual angiogenic and immunosuppressive roles of vascular endothelial growth factor (VEGF), which bevacizumab targets to complement atezolizumab [9].

In external validation, CAPTYN achieved an AUC of 0.70 (95% CI, 0.59 to 0.81) in IMbrave150, significantly outperforming CRAFITY and ALBI (DeLong *P* < 0.05; Fig. 1A and Table S5). CRAPT-M could not be evaluated in IMbrave150 because PIVKA-II was not available in this cohort. CAPTYN also showed the best calibration, with the lowest Brier score (Fig. 1A and Table S6). In survival analyses, all models significantly stratified OS; however, only CAPTYN significantly stratified PFS (Fig. 1B). Results were consistent in the real-world validation cohort (YUHS): CAPTYN achieved an AUC of 0.67 (95% CI, 0.59 to 0.75) and the lowest Brier score (Fig. S10 and Tables S5 and S6). Only CAPTYN significantly stratified both OS and PFS, whereas CRAFITY and CRAPT-M stratified OS only. Across both validation cohorts, CAPTYN showed balanced binary classification metrics (Fig. S11) and higher net benefit on decision curve analysis across thresholds of 0.3 to 0.6 (Fig. S12). In IMbrave150, multivariate Cox regression adjusting for covariates including Child–Pugh score, ECOG PS, and PT-INR confirmed CAPTYN as an independent prognostic factor for both OS (hazard ratio [HR] 2.57; *P* < 0.001) and PFS (HR 2.12; *P* = 0.002) (Table S7). Subgroup analyses demonstrated consistent prognostic stratification, with HRs ≥ 1.49 for nonclinical benefit across all strata and a significant effect in the Western subgroup (OS and PFS, *P* = 0.003 each) (Fig. 1C).

**Fig. 1.**
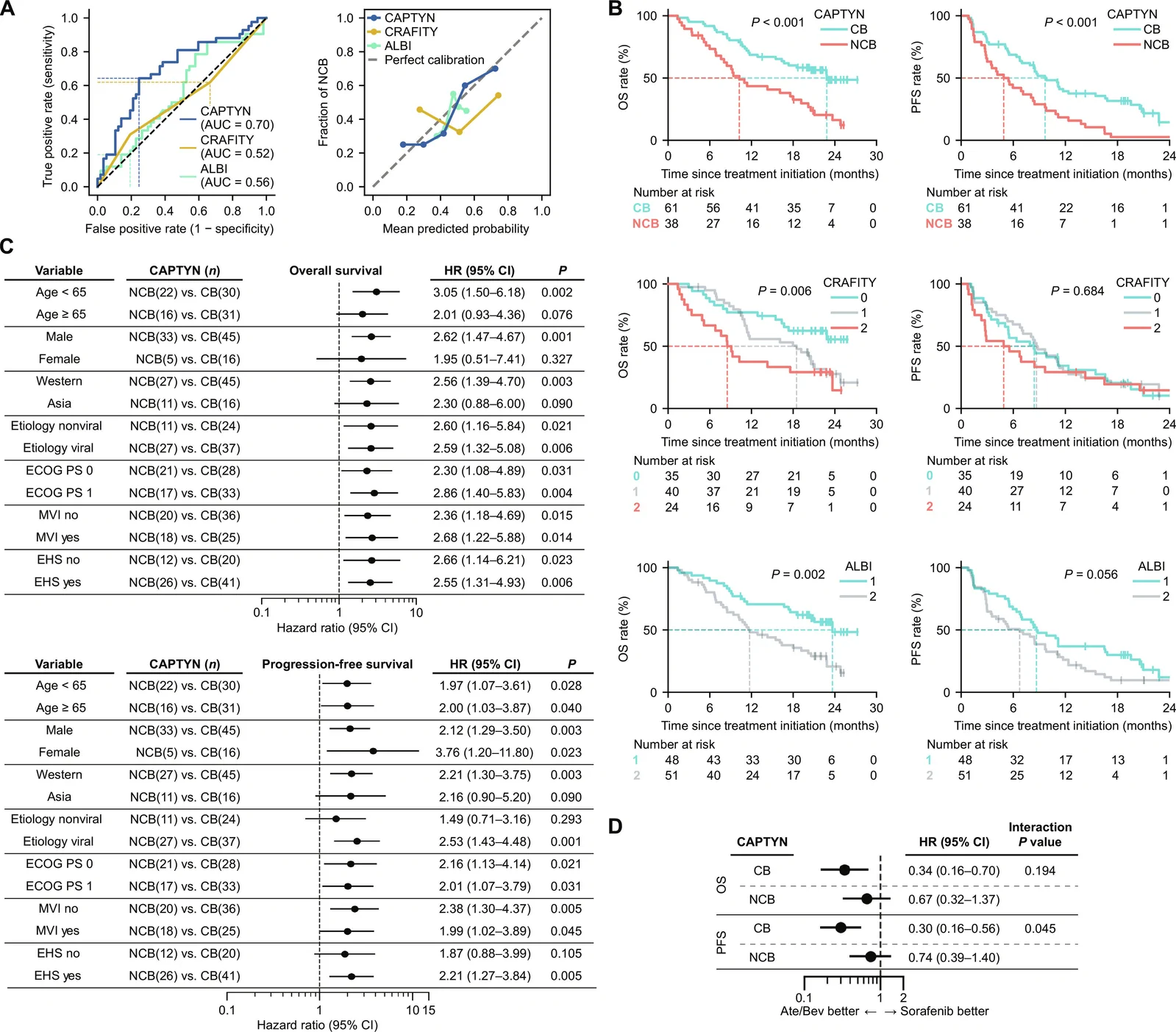
External validation and treatment-specific evaluation of CAPTYN in advanced hepatocellular carcinoma. (A) Receiver operating characteristic curves (left) and calibration plots (right) comparing CAPTYN, CRAFITY, and ALBI for prediction of nonclinical benefit in the IMbrave150 cohort. In the receiver operating characteristic curves, the diagonal dashed line indicates the line of no discrimination, and the thin dashed lines mark the sensitivity and false positive rate at each model’s reference cutoff (CAPTYN ≥ 0.486, CRAFITY > 1, and ALBI ≥ −2.27). (B) Kaplan–Meier curves of OS and PFS stratified by CAPTYN (top), CRAFITY (middle), and ALBI (bottom) in the IMbrave150 cohort. CRAFITY scores of 0, 1, and 2 indicate the number of risk factors present (CRP ≥ 1 mg/dL and AFP ≥ 100 ng/mL). ALBI 1 and 2 indicate ALBI grades 1 (score ≤ −2.60) and 2 (score > −2.60 to ≤ −1.39), respectively. Dashed lines indicate median survival. (C) Subgroup analysis of OS (top) and PFS (bottom) in the IMbrave150 cohort. Hazard ratios for nonclinical benefit versus clinical benefit are shown across clinical subgroups. (D) Treatment–prediction interaction for OS and PFS in the IMbrave150 trial. Hazard ratios for Ate/Bev versus sorafenib are shown separately for CAPTYN-predicted clinical benefit and nonclinical benefit groups, along with the *P* value for interaction. Abbreviations: AFP, α-fetoprotein; Ate/Bev, atezolizumab plus bevacizumab; AUC, area under the curve; CB, clinical benefit; CI, confidence interval; CRP, C-reactive protein; ECOG PS, Eastern Cooperative Oncology Group performance status; EHS, extrahepatic spread; HR, hazard ratio; MVI, macrovascular invasion; NCB, nonclinical benefit; OS, overall survival; PFS, progression-free survival.

To explore the Ate/Bev specificity of CAPTYN, we examined TKI-treated cohorts. In both the SOR and LEN cohorts, CAPTYN did not stratify OS or PFS, and its discrimination did not extend to TKI response, whereas general prognostic scores (CRAFITY and/or ALBI) retained survival stratification (AUC was not estimable in the SOR cohort owing to only 2 clinical benefit events; Figs. S13 to S15, DeLong test in Table S8). Consistent with this, in IMbrave150, the PFS benefit of Ate/Bev over sorafenib was greater in CAPTYN-predicted clinical benefit than nonclinical benefit patients (interaction *P* = 0.045 for PFS but not OS [*P* = 0.194]; Fig. 1D). These exploratory findings are consistent with CAPTYN capturing biology relevant to immune–VEGF combination therapy.

This study had several limitations. The small TKI control cohorts (SOR, *n* = 28) and the lack of external validation for other first-line regimens (tremelimumab plus durvalumab and nivolumab plus ipilimumab) limited treatment-specific inference. Although external validation showed moderate discrimination (AUC 0.67 to 0.70), this may partly reflect the inability of blood-based measurements to capture intrapatient heterogeneity; integrating radiomic and molecular features may further enhance predictions [10]. Prospective, multiregional validation is needed to confirm clinical utility.

In conclusion, CAPTYN is an externally validated machine-learning model that predicts clinical outcomes with Ate/Bev in advanced HCC using 6 routinely available laboratory variables. CAPTYN demonstrated consistent discrimination, calibration, and survival stratification across IMbrave150 and an independent real-world cohort, outperforming established prognostic scores. Its treatment specificity awaits validation in larger randomized trials, but CAPTYN may serve as a practical prognostic tool for risk stratification in advanced HCC.

## Ethical Approval

This study was approved by the Institutional Review Board of the affiliated universities (CHA Bundang Medical Center: 2023-03-054; Medical University of Vienna: 2033/2017; Yonsei University Health System: 4-2023-1273) and was conducted in accordance with the Declaration of Helsinki (2024). The requirement for written informed consent was waived due to the retrospective study design.

## Data Availability

The data that support the findings of this study are not publicly available because they contain information that could compromise the privacy of research participants. The R and Python code for reproducing the results is available upon reasonable request from the corresponding authors, and the model hyperparameter settings are listed in Table S9. The CAPTYN score calculator is available as a macro-enabled Excel file (.xlsm) (Supplementary Material 2). An online CAPTYN calculator is also available at https://cha-captyn.streamlit.app.

## Supplementary Materials

Supplementary 1 Supplementary Methods Figs. S1 to S15 Tables S1 to S9 TRIPOD+AI Checklist Supplementary Material 2
