# Supplementary material for: CAPTYN, a Blood-Based Machine-Learning Model Predicting Clinical Benefit of Atezolizumab–Bevacizumab in Hepatocellular Carcinoma: Validation in the IMbrave150 Trial
Source: Cancer Commun (Lond). 2026 Oct 19;46:0059. doi: 10.34133/cancomm.0059 (PMC13678081; doi:10.34133/cancomm.0059)
Supplement: Supplementary file 1 [file cancomm.0059.f1.zip › CANCOMM-D-26-00521_suppleMat_1_final.pdf]

Supplementary Materials for

CAPTYN, a Blood-Based Machine-Learning Model Predicting Clinical Benefit of Atezolizumab–Bevacizumab in Hepatocellular Carcinoma: Validation in IMbrave150 Trial

Gae Hoon Jo[1†], Sohyun Hwang[2†], Bernhard Scheiner[3,4†], Seonjeong Woo[1], Won Suk Lee[5], Beodeul Kang[5], Jung Sun Kim[5], Ho Yeong Lim[5], Chansik An[6], Dong Yun Kim[7,8], Inyoung Kim[9], Dong-hyuk Heo[9], Matthias Pinter[3,4*], Beom Kyung Kim[7,8*], Chan Kim[10*], and Hong Jae Chon[5*]

[1]Department of Life Science, Graduate School, CHA University, Seongnam, Republic of Korea.
[2]Department of Pathology, CHA Bundang Medical Center, CHA University School of Medicine, Seongnam, Republic of Korea.
[3]Division of Gastroenterology and Hepatology, Department of Medicine III, Medical University of Vienna, Vienna, Austria.
[4]Vienna Liver Cancer Study Group, Division of Gastroenterology and Hepatology, Department of Medicine III, Medical University of Vienna, Vienna, Austria.
[5]Department of Medical Oncology, CHA Bundang Medical Center, CHA University School of Medicine, Seongnam, Republic of Korea.
[6]Department of Radiology, CHA Bundang Medical Center, CHA University School of Medicine, Seongnam, Republic of Korea.
[7]Department of Internal Medicine, Yonsei University College of Medicine, Seoul, Republic of Korea.
[8]Yonsei Liver Center, Severance Hospital, Seoul, Republic of Korea.
[9]Theragen Bio Co., Ltd., Seongnam, Republic of Korea.
[10]Department of Biochemistry and Molecular Biology, Graduate School of Medical Science, Brain Korea 21 Project, Yonsei University College of Medicine, Seoul, Republic of Korea.

*Address correspondence to: H.J.C. (Hong Jae Chon), minidoctor@cha.ac.kr; C.K. (Chan Kim), chankim@yuhs.ac; B.K.K. (Beom Kyung Kim), beomkkim@yuhs.ac; M.P. (Matthias Pinter), matthias.pinter@meduniwien.ac.at.

†These authors contributed equally to this study.

Supplementary Materials include

Supplementary Material 1:

Supplementary Methods

Figures S1 to S15

Tables S1 to S9

TRIPOD+AI Checklist

Supplementary Material 2:

CAPTYN score calculator (Excel file)

Supplementary Methods

Study design and patients

This multicenter study adhered to the TRIPOD+AI statement [1]. We screened 949 patients with hepatocellular carcinoma (HCC) from three hospitals and one randomized trial and grouped them by first-line regimen into four atezolizumab plus bevacizumab (Ate/Bev)-treated cohorts and two tyrosine kinase inhibitor (TKI)-treated cohorts (**Fig. S1**). The inclusion criteria were unresectable HCC, age ≥18 years, Eastern Cooperative Oncology Group performance status (ECOG PS) ≤1, Barcelona Clinic Liver Cancer (BCLC) stage B/C, Child–Pugh class A/B, and Ate/Bev or TKI as first-line therapy. The exclusion criteria were hepatitis B and C virus (HBV and HCV) coinfection, missing values for any baseline variables used in the analysis, or missing overall survival (OS), progression-free survival (PFS), or Response Evaluation Criteria in Solid Tumors version 1.1 (RECIST v1.1) best overall response. After exclusion, all eligible patients (n = 737) were analyzed. This study was approved by the Institutional Review Board of the affiliated universities (approval number for CHA Bundang Medical Center: 2023-03-054; approval number for Medical University of Vienna: 2033/2017; approval number for Yonsei University Health System: 4-2023-1273) and was conducted in accordance with the Declaration of Helsinki (2024). The requirement for written informed consent was waived due to the retrospective study design.

For model development, the training set comprised Ate/Bev-treated patients from the CHA Bundang Medical Center, Korea (CHA; n = 301; June 2020–October 2023) and the Medical University of Vienna, Austria (Vienna; n = 53; February 2020–November 2024). The external validation set included Ate/Bev-treated patients from the prospective phase III IMbrave150 trial (IMbrave150; n = 99; March 2018–April 2019; NCT03434379) and the Yonsei University Health System, Korea (YUHS; n = 184; September 2018–August 2023). To evaluate treatment specificity, the control set comprised lenvatinib-treated patients from YUHS, Korea (LEN; n = 72; October 2018–January 2024) and sorafenib-treated patients from IMbrave150 (SOR; n = 28; March 2018–April 2019). In the IMbrave150 trial, 140 Ate/Bev-treated and 58 sorafenib-treated patients were initially available. After excluding patients with missing baseline clinical variables, 99 Ate/Bev-treated patients were included in the external validation set, and 28 sorafenib-treated patients were included in the control set (**Fig. S1**). No formal sample size calculation was performed, given the retrospective nature of the analyses.

Outcome definitions

Patients were classified as having clinical benefit (CB) or non-clinical benefit (NCB). CB was defined as a complete response, partial response, or stable disease (SD) with PFS ≥6 months according to RECIST v1.1, consistent with the Society for Immunotherapy of Cancer guidance [2]. All other patients, including those with SD with PFS <6 months or progressive disease, were classified as NCB.

Predictor selection and model development

Eighteen baseline clinical variables were recorded at treatment initiation. The overall flowchart for model development is displayed in **Fig. S2**. Protein induced by vitamin K absence or antagonist-II (PIVKA-II) was excluded due to limited availability across cohorts, and Child–Pugh class and ECOG PS were excluded to avoid including subjective composite variables [3]. Prothrombin time–international normalized ratio (PT-INR) was excluded as redundant with the routine liver-function markers albumin and total bilirubin: it was correlated with both (**Fig. S3A, B**) and was not independently associated with OS or PFS when modeled together with them (**Fig. S3C**). Removing such redundant inputs reduces collinearity and improves the parsimony and stability of feature selection. This left 14 variables, which we evaluated using univariate Cox regression analyses for OS and PFS (**Table S3**); variables significant for either OS or PFS were selected as candidate predictors. Next, recursive feature elimination (RFE) was used to determine the final predictor set. To select the classifier for RFE, we compared four algorithms: eXtreme Gradient Boosting (XGBoost), random forest, support vector classifier (SVC), and logistic regression (LR), based on 5-fold cross-validated (CV) area under the curve (AUC) for NCB classification (**Fig. S4**). Because SVC and LR are scale-sensitive, predictors were z-standardized within each training fold. Hyperparameters for each classifier were optimized within predefined search spaces (**Table S9**). Among the four algorithms, XGBoost achieved the highest mean CV AUC and was therefore selected as the primary classifier for RFE. RFE then iteratively removed one variable at a time by excluding the variable whose removal produced the largest improvement in 5-fold CV AUC for NCB classification, continuing until two variables remained (**Fig. S5**). The variable set yielding the highest AUC was selected as the final six-variable predictor set. Using this predictor set, we trained the final XGBoost model on the entire training cohort with optimized hyperparameters (**Fig. S6**). The final hyperparameters were subsample = 0.5, reg_alpha = 0, and reg_lambda = 1, with all others (including max_depth = 6 and learning rate = 0.3) left at default.

Model benchmarking and evaluation

We evaluated the performance of the developed model by benchmarking it against existing prognostic models, including CRAFITY (C-reactive protein [CRP] and α-fetoprotein [AFP] in immunotherapy), CRAPT-M (CRP, albumin, PIVKA-II, total bilirubin, and macrovascular invasion), and ALBI (albumin–bilirubin). Based on prior studies, binary thresholds for NCB were defined as CRAFITY > 1, CRAPT-M > 12, and ALBI ≥ −2.27 [4–6]. For survival stratification, patients were grouped by CRAFITY score (0, 1, or 2), CRAPT-M risk group (low, ≤4; intermediate, 5–12; high, ≥13), and ALBI grade (1, 2, or 3) [3]. The classification threshold for the developed model was determined using Youden's *J* in the training set (**Fig. S7**). Model performance was evaluated using the AUC, with DeLong's test for comparison, as well as accuracy, F1 score, sensitivity, specificity, positive predictive value, and negative predictive value. For calibration and decision curve analysis,

the CRAFITY and ALBI scores were converted to predicted probabilities of NCB using logistic regression, and CRAPT-M was excluded due to the limited availability of PIVKA-II. Calibration was summarized using the Brier score and five-bin calibration plots.

Model interpretation

Model interpretability was assessed using the SHapley Additive exPlanations (SHAP) framework [7]. SHAP values quantify the marginal contribution of each variable to the model's prediction, accounting for potential variable interactions. In this study, with NCB defined as the positive class, positive SHAP values (>0) indicated a contribution toward NCB prediction, while negative values (<0) favored CB.

Statistical analysis

Survival outcomes were evaluated using the Kaplan–Meier method with log-rank tests, and univariate and multivariate analyses were conducted using Cox proportional hazards models. The median follow-up duration was calculated using the reverse Kaplan–Meier method. Treatment-specific predictive effects were assessed by fitting Cox models that included the model-predicted class, treatment group, and their interaction, with treatment prediction specificity evaluated using the interaction *P*-value. Subgroup analyses across demographic and clinical strata were performed using Cox regression. To evaluate the potential bias arising from missing radiological response data, we performed a worst-case sensitivity analysis in which all missing values were imputed as disease progression (NCB).

All survival analyses were conducted in Python using the lifelines package (v0.30.0). Data preprocessing was performed using pandas (v2.3.1) and numpy (v2.2.6) in Python and dplyr (v1.1.4) in R. Model development, comparison, and performance evaluation were performed using scikit-learn (v1.7.0) and xgboost (v3.0.2) in Python. Logistic regression analyses were performed using the statsmodels package (v0.14.5) in Python. Visualizations were generated using Matplotlib (v3.10.3) in Python and ComplexHeatmap (v2.24.0) in R. All analyses were performed using Python (v3.10.18) and R (v4.5.0).

References

1. Collins GS, Moons KGM, Dhiman P, Riley RD, Beam AL, Van Calster B, Ghassemi M, Liu X, Reitsma JB, van Smeden M, et al. TRIPOD+AI statement: updated guidance for reporting clinical prediction models that use regression or machine learning methods. BMJ. 2024;385:e078378.
2. Kluger HM, Tawbi HA, Ascierto ML, Bowden M, Callahan MK, Cha E, Chen HX, Drake CG, Feltquate DM, Ferris RL, et al. Defining tumor resistance to PD-1 pathway blockade: recommendations from the first meeting of the SITC Immunotherapy Resistance Taskforce. J Immunother Cancer. 2020;8(1):e000398.

3. Johnson PJ, Berhane S, Kagebayashi C, Satomura S, Teng M, Reeves HL, O'Beirne J, Fox R, Skowronska A, Palmer D, et al. Assessment of liver function in patients with hepatocellular carcinoma: a new evidence-based approach-the ALBI grade. J Clin Oncol. 2015;33(6):550–558.
4. Scheiner B, Pomej K, Kirstein MM, Hucke F, Finkelmeier F, Waidmann O, Himmelsbach V, Schulze K, von Felden J, Fründt TW, et al. Prognosis of patients with hepatocellular carcinoma treated with immunotherapy - development and validation of the CRAFITY score. J Hepatol. 2022;76(2):353–363.
5. Hiraoka A, Kumada T, Tsuji K, Takaguchi K, Itobayashi E, Kariyama K, Ochi H, Tajiri K, Hirooka M, Shimada N, et al. Validation of Modified ALBI Grade for More Detailed Assessment of Hepatic Function in Hepatocellular Carcinoma Patients: A Multicenter Analysis. Liver Cancer. 2019;8(2):121–129.
6. Nam H, Kim DY, Kim DY, Kim JH, Kim CW, Lee J, Yang K, Han JW, Sung PS, Yoon SK, et al. Development and validation of a risk prediction model for patients with hepatocellular carcinoma receiving atezolizumab-bevacizumab. Hepatology. 2026;83(5):1098–1110.
7. Lundberg SM, Erion G, Chen H, DeGrave A, Prutkin JM, Nair B, Katz R, Himmelfarb J, Bansal N, Lee SI. From Local Explanations to Global Understanding with Explainable AI for Trees. Nat Mach Intell. 2020;2(1):56–67.

Supplementary Figures

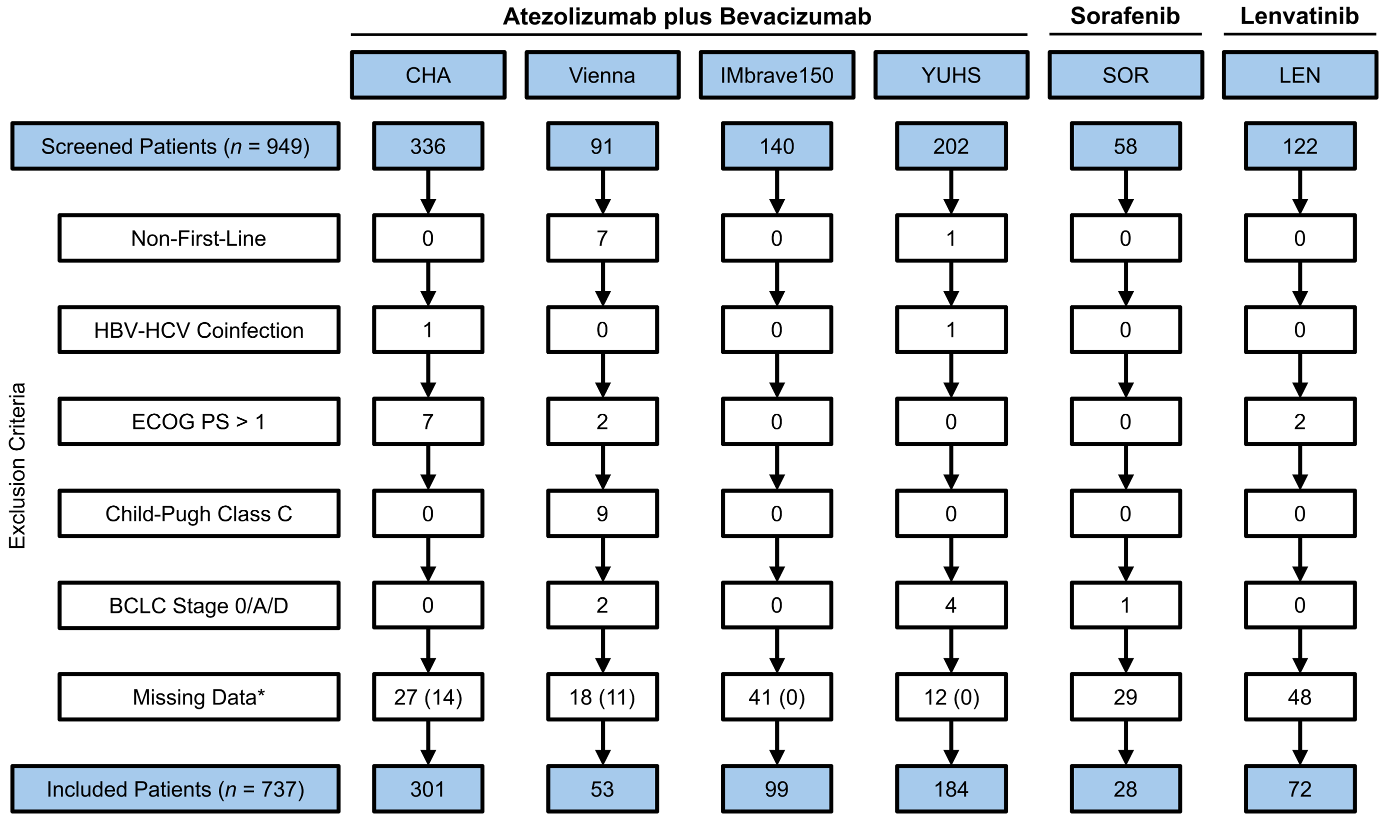

Fig. S1. Patient flowchart. Numbers in boxes indicate the number of patients at each step. *Missing data were defined as missing values in study variables, survival information, or tumor response evaluation. Numbers in parentheses denote patients with missing tumor response data who were included in the sensitivity analysis. BCLC, Barcelona Clinic Liver Cancer; CHA, CHA Bundang Medical Center; ECOG PS, Eastern Cooperative Oncology Group performance status; HBV, Hepatitis B virus; HCV, Hepatitis C virus; LEN, lenvatinib-treated cohort; SOR, sorafenib-treated cohort; YUHS, Yonsei University Health System.

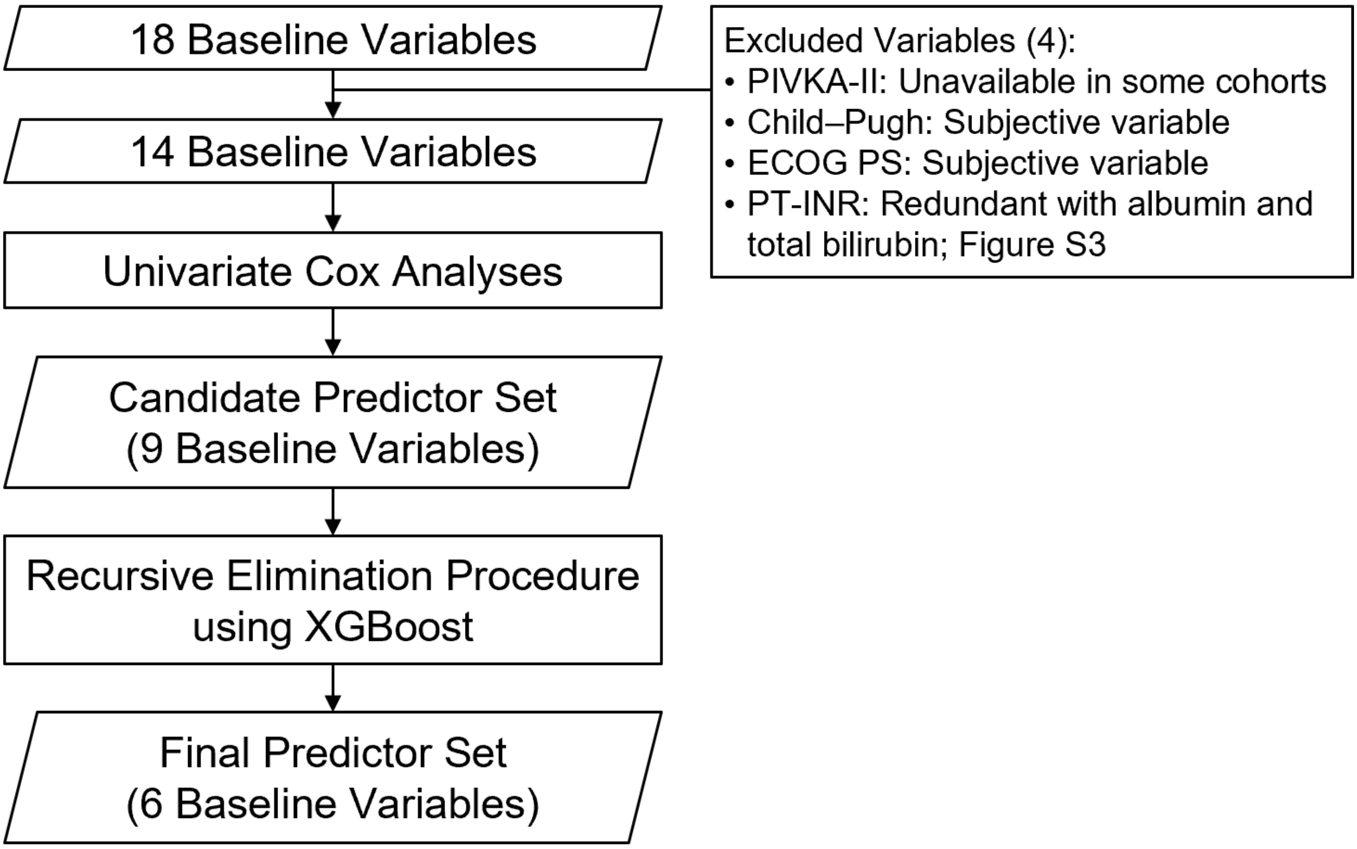

Fig. S2. Model development flowchart. ECOG PS, Eastern Cooperative Oncology Group performance status; PIVKA-II, protein induced by vitamin K absence or antagonist-II; PT-INR, prothrombin time–international normalized ratio.

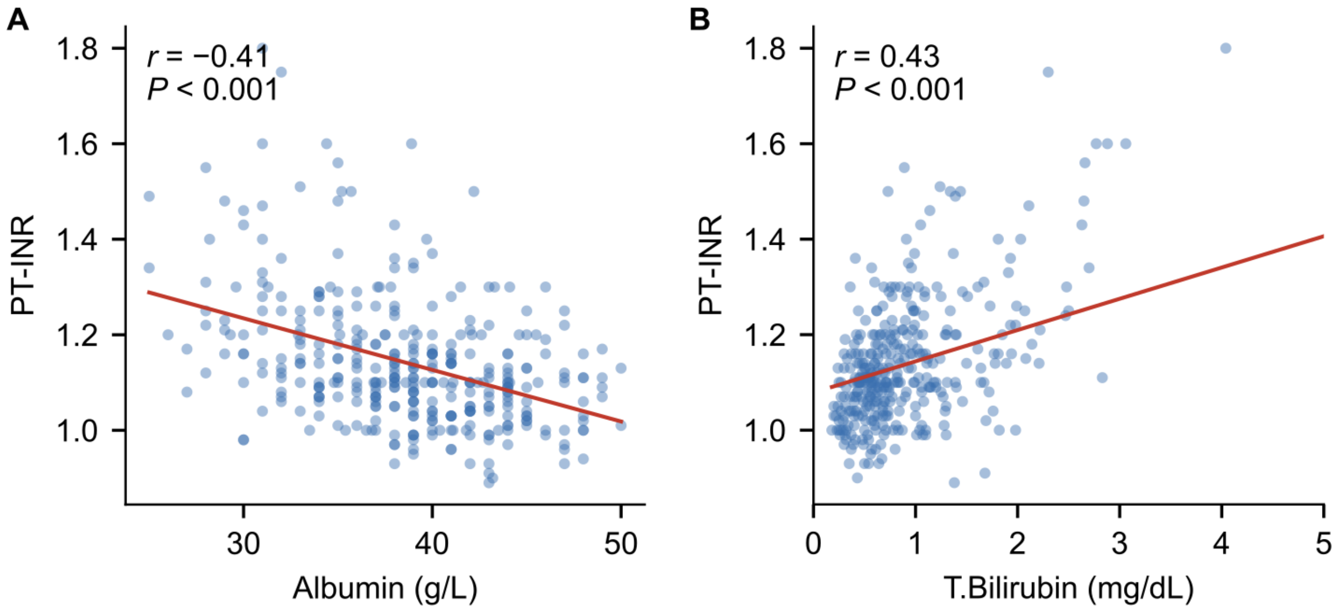

C

Variables	Overall Survival		Progression-Free Survival	
	Hazard Ratio (95% CI)	*P*	Hazard Ratio (95% CI)	*P*
Albumin (g/L)	0.94 (0.91–0.96)	< 0.001	0.99 (0.97–1.02)	0.469
T.Bilirubin (mg/dL)	1.33 (1.18–1.50)	< 0.001	1.27 (1.10–1.47)	< 0.001
PT-INR	1.26 (0.44–3.62)	0.669	1.54 (0.57–4.20)	0.395

Fig. S3. Redundancy of PT-INR with albumin and total bilirubin in the training set (*n* = 354). (A) Scatterplots of PT-INR against albumin and **(B)** total bilirubin; PT-INR was significantly correlated with both markers. **(C)** Multivariate Cox regression of the three markers for overall survival and progression-free survival. PT-INR was not independently associated when modeled together with albumin and total bilirubin, whereas total bilirubin remained associated with both. CI, confidence interval; PT-INR, prothrombin time–international normalized ratio; T.Bilirubin, total bilirubin.

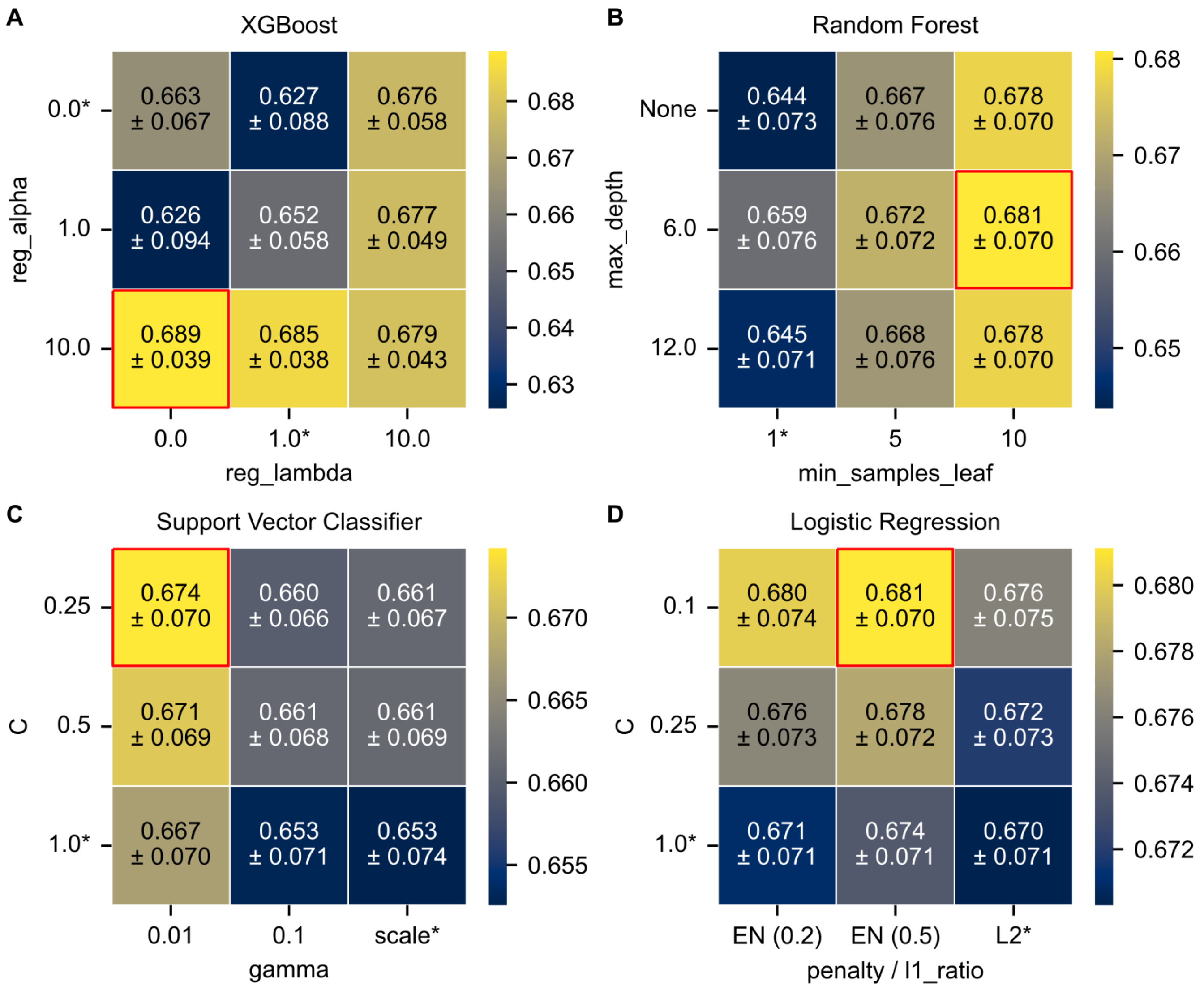

Fig. S4. Comparison of machine-learning models for NCB classification in the training set. Hyperparameter search grids are shown for **(A)** XGBoost, **(B)** Random Forest, **(C)** Support Vector Classifier, and **(D)** Logistic Regression. Default values are indicated by an asterisk (*). The mean 5-fold cross-validated AUC with standard deviation is shown in each grid cell, and values were examined to three decimal places to identify the best-performing condition; the best-performing hyperparameter combination for each model is highlighted in red. AUC, area under the curve; EN, Elastic Net; NCB, non-clinical benefit.

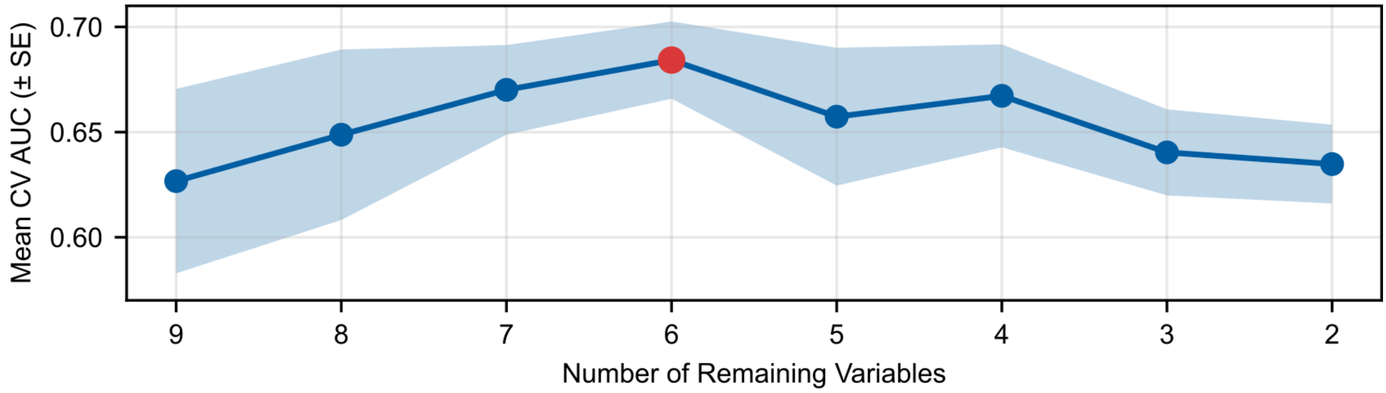

No. of Variables	Variables	Mean CV AUC (± SE)
9	Platelet, CRP, AFP, Lymphocyte, T.Bilirubin, Neutrophil, MVI, Age, Albumin	0.63 ± 0.04
8	Platelet, CRP, AFP, Lymphocyte, T.Bilirubin, Neutrophil, MVI, Age	0.65 ± 0.04
7	Platelet, CRP, AFP, Lymphocyte, T.Bilirubin, Neutrophil, MVI	0.67 ± 0.02
**6**	**Platelet, CRP, AFP, Lymphocyte, T.Bilirubin, Neutrophil**	**0.68 ± 0.02**
5	Platelet, CRP, AFP, Lymphocyte, T.Bilirubin	0.66 ± 0.03
4	Platelet, CRP, AFP, Lymphocyte	0.67 ± 0.02
3	Platelet, CRP, AFP	0.64 ± 0.02
2	Platelet, CRP	0.63 ± 0.02

Fig. S5. Recursive feature elimination identifies the optimal predictor set for the XGBoost classifier in the training set. The mean 5-fold CV AUC for NCB classification is plotted against the number of variables retained during recursive feature elimination; shaded bands represent ± 1 standard error. Peak performance was achieved with six variables, which were selected as the final predictor set. The accompanying table lists representative subsets evaluated during elimination, with the selected six-variable model shown in bold. AFP, α-fetoprotein; AUC, area under the curve; CRP, C-reactive protein; CV, cross-validation; MVI, macrovascular invasion; NCB, non-clinical benefit; SE, standard error; T.Bilirubin, total bilirubin.

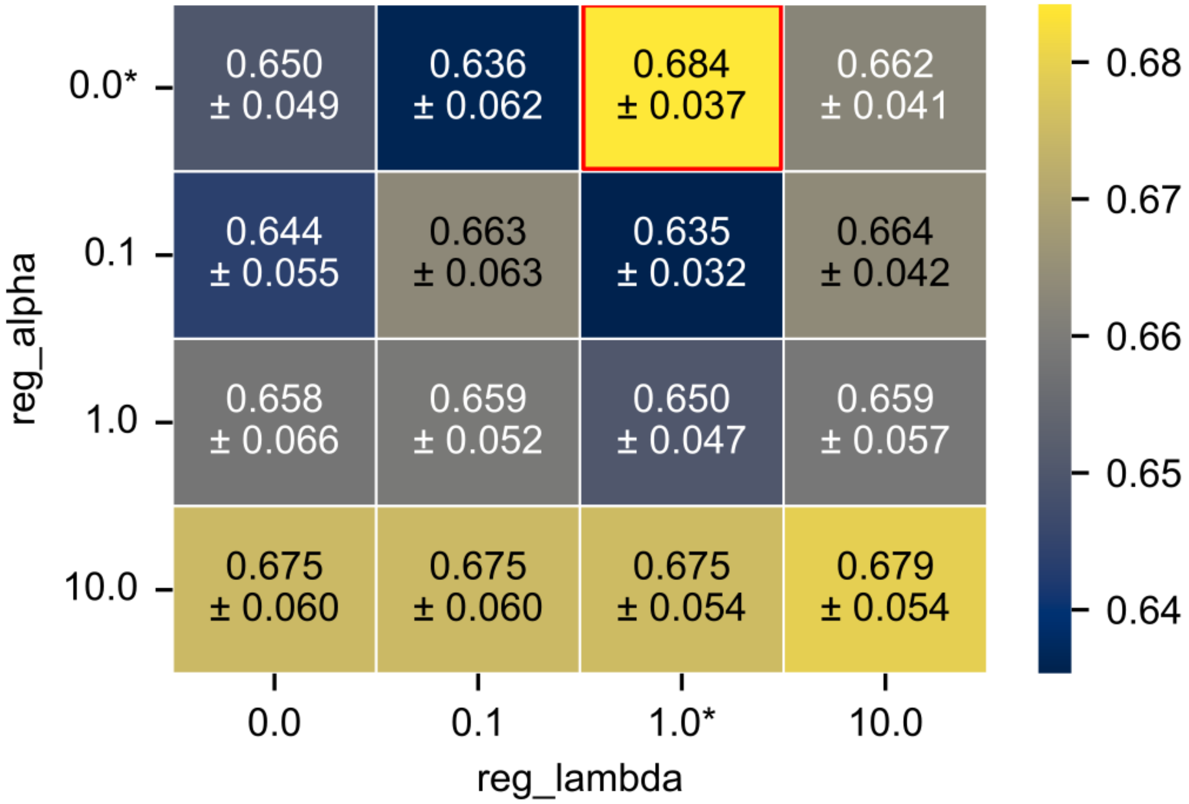

Fig. S6. Hyperparameter optimization of the final XGBoost classifier for NCB classification in the training set. Heatmap of mean 5-fold cross-validated AUC values (with standard deviation) across the predefined hyperparameter search space for the XGBoost classifier trained on the final six-variable predictor set. The regularization terms (reg_alpha and reg_lambda) were searched over a finer grid (0, 0.1, 1, and 10) than the grid used for the initial algorithm comparison in **Table S9**. Default values are indicated by asterisks (*). Values are shown to three decimal places. The best-performing hyperparameter combination is highlighted in red. AUC, area under the curve; NCB, non-clinical benefit.

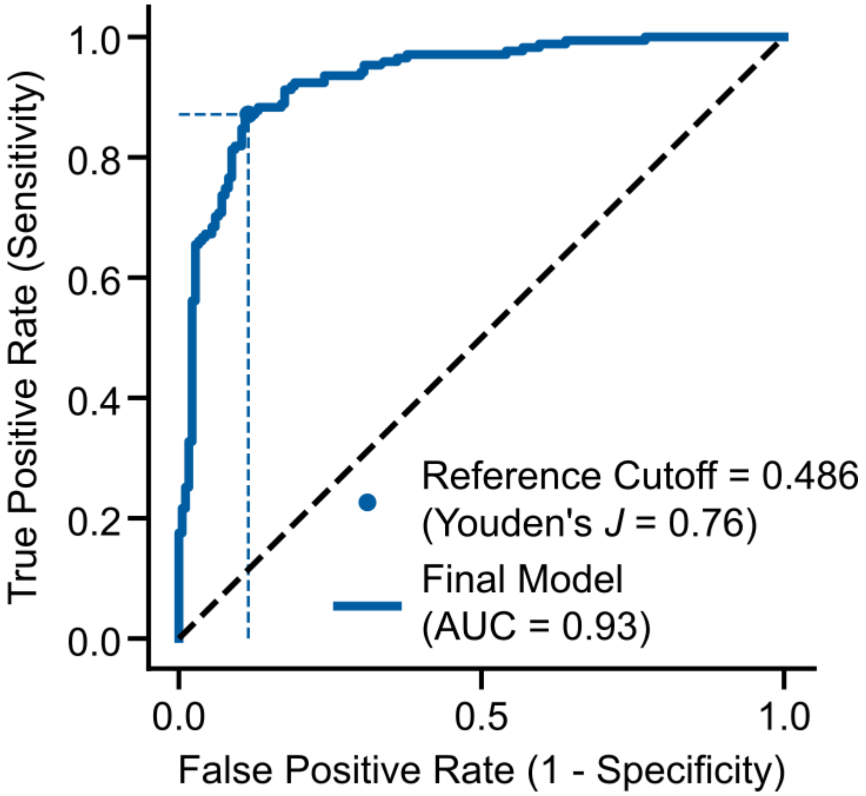

Fig. S7. Receiver operating characteristic curve of the final XGBoost model in the training set. The ROC curve illustrates the discriminative performance of the final XGBoost model for predicting NCB in the training cohort (n = 354). A reference classification cutoff of 0.486 was derived using Youden's J statistic. AUC, area under the curve; NCB, non-clinical benefit; ROC, receiver operating characteristic.

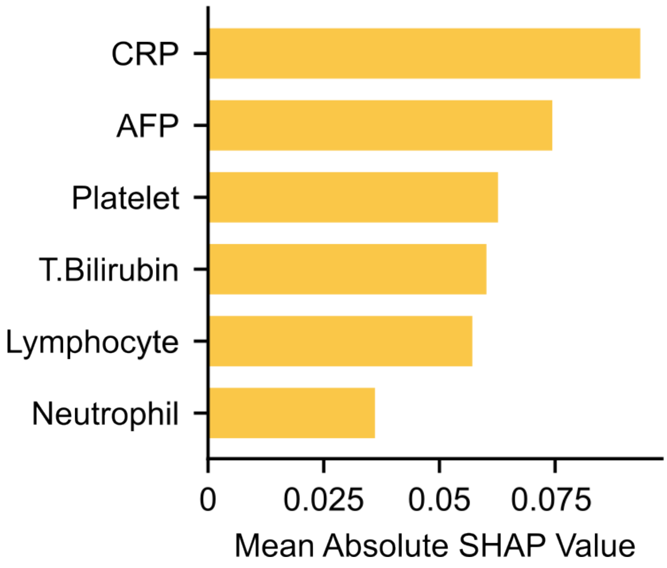

Fig. S8. Feature importance of the six predictors in the final XGBoost model. Bars represent the mean absolute SHAP values of each predictor in the training set, ranked by decreasing contribution. Higher values indicate greater overall impact on non-clinical benefit prediction. AFP, α-fetoprotein; CRP, C-reactive protein; SHAP, Shapley additive explanations; T.Bilirubin, total bilirubin.

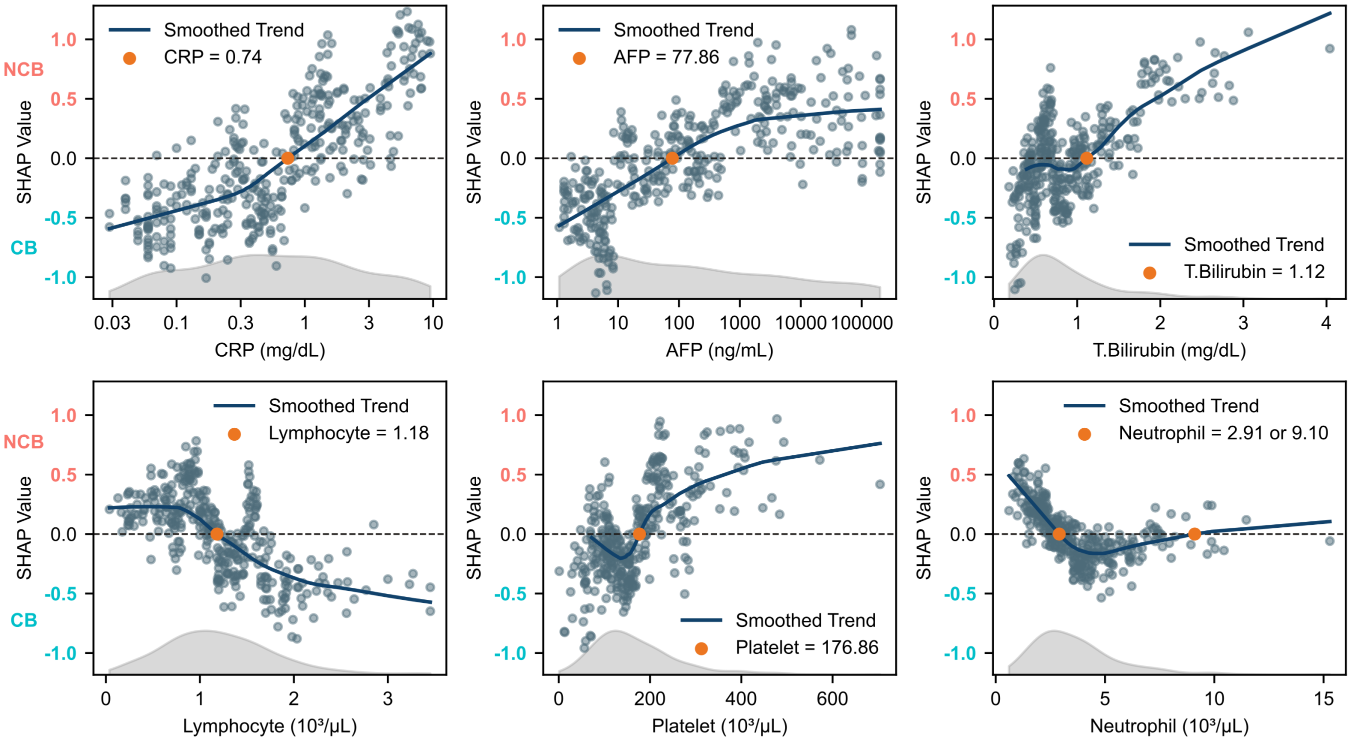

Fig. S9. SHAP dependence plots for the six predictors in the CAPTYN model. Each panel shows the SHAP value of one predictor against its observed value in the training cohort. Positive SHAP values (>0) indicate a contribution toward NCB prediction, whereas negative values (<0) favor CB. Smoothed trend lines indicate the direction of effect, and orange dots mark threshold values where the fitted curve crosses SHAP = 0. AFP, α-fetoprotein; CB, clinical benefit; CRP, C-reactive protein; NCB, non-clinical benefit; SHAP, Shapley additive explanations; T.Bilirubin, total bilirubin.

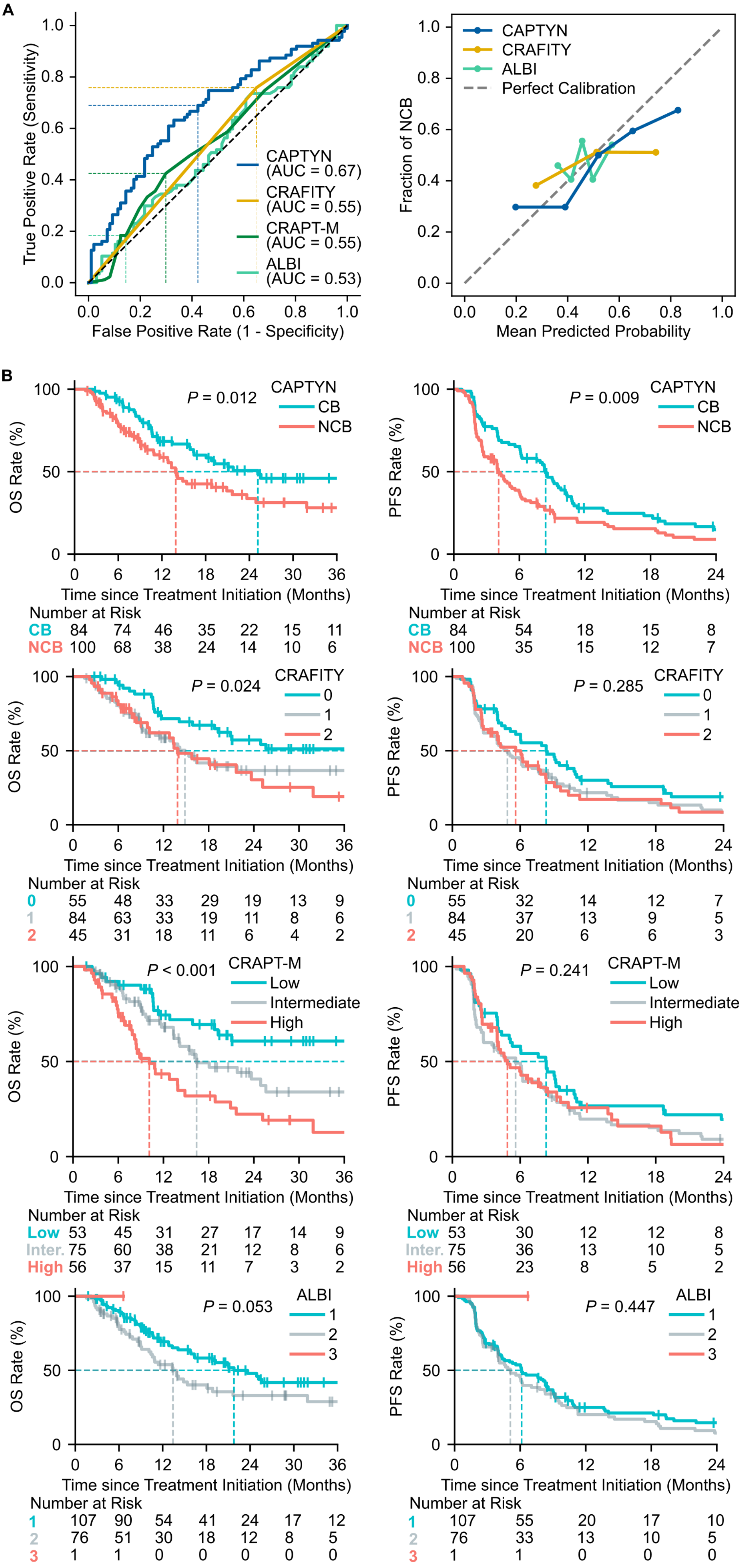
A
True Positive Rate (Sensitivity)
False Positive Rate (1 - Specificity)
CAPTYN (AUC = 0.67)
CRAFITY (AUC = 0.55)
CRAPT-M (AUC = 0.55)
ALBI (AUC = 0.53)
Fraction of NCB
Mean Predicted Probability
CAPTYN
CRAFITY
ALBI
Perfect Calibration
B
OS Rate (%)
PFS Rate (%)
Time since Treatment Initiation (Months)
P = 0.012
CAPTYN
CB
NCB
Number at Risk
CB 84 74 46 35 22 15 11
NCB 100 68 38 24 14 10 6
P = 0.009
CAPTYN
CB
NCB
Number at Risk
CB 84 54 18 15 8
NCB 100 35 15 12 7
P = 0.024
CRAFITY
0
1
2
Number at Risk
0 55 48 33 29 19 13 9
1 84 63 33 19 11 8 6
2 45 31 18 11 6 4 2
P = 0.285
CRAFITY
0
1
2
Number at Risk
0 55 32 14 12 7
1 84 37 13 9 5
2 45 20 6 6 3
P < 0.001
CRAPT-M
Low
Intermediate
High
Number at Risk
Low 53 45 31 27 17 14 9
Inter. 75 60 38 21 12 8 6
High 56 37 15 11 7 3 2
P = 0.241
CRAPT-M
Low
Intermediate
High
Number at Risk
Low 53 30 12 12 8
Inter. 75 36 13 10 5
High 56 23 8 5 2
P = 0.053
ALBI
1
2
3
Number at Risk
1 107 90 54 41 24 17 12
2 76 51 30 18 12 8 5
3 1 1 0 0 0 0 0
P = 0.447
ALBI
1
2
3
Number at Risk
1 107 55 20 17 10
2 76 33 13 10 5
3 1 1 0 0 0

Fig. S10. External validation of CAPTYN compared with existing prognostic models in the real-world Korean cohort (YUHS). (A) ROC curves (left) and calibration plots (right) for prediction of NCB in the YUHS cohort (n = 184). CRAPT-M was excluded from calibration analysis due to the limited availability of protein induced by vitamin K absence or antagonist-II. In the ROC curves, the diagonal dashed line indicates the line of no discrimination, and the thin dashed lines mark the sensitivity and false positive rate at each model's reference cutoff (CAPTYN ≥ 0.486, CRAFITY > 1, CRAPT-M > 12, and ALBI ≥ −2.27). **(B)** Kaplan–Meier curves of OS and PFS stratified by CAPTYN (top), CRAFITY (second row), CRAPT-M (third row), and ALBI (bottom). CRAFITY scores of 0, 1, and 2 indicate the number of risk factors present (CRP ≥ 1 mg/dL and AFP ≥ 100 ng/mL). CRAPT-M risk groups were defined as low (score ≤ 4), intermediate (5–12), and high (≥ 13). ALBI 1, 2, and 3 indicate ALBI grades 1 (score ≤ −2.60), 2 (score > −2.60 to ≤ −1.39), and 3 (score > −1.39), respectively. Dashed lines indicate median survival. AFP, α-fetoprotein; AUC, area under the curve; CB, clinical benefit; CRP, C-reactive protein; Inter., intermediate; NCB, non-clinical benefit; OS, overall survival; PFS, progression-free survival; ROC, receiver operating characteristic; YUHS, Yonsei University Health System.

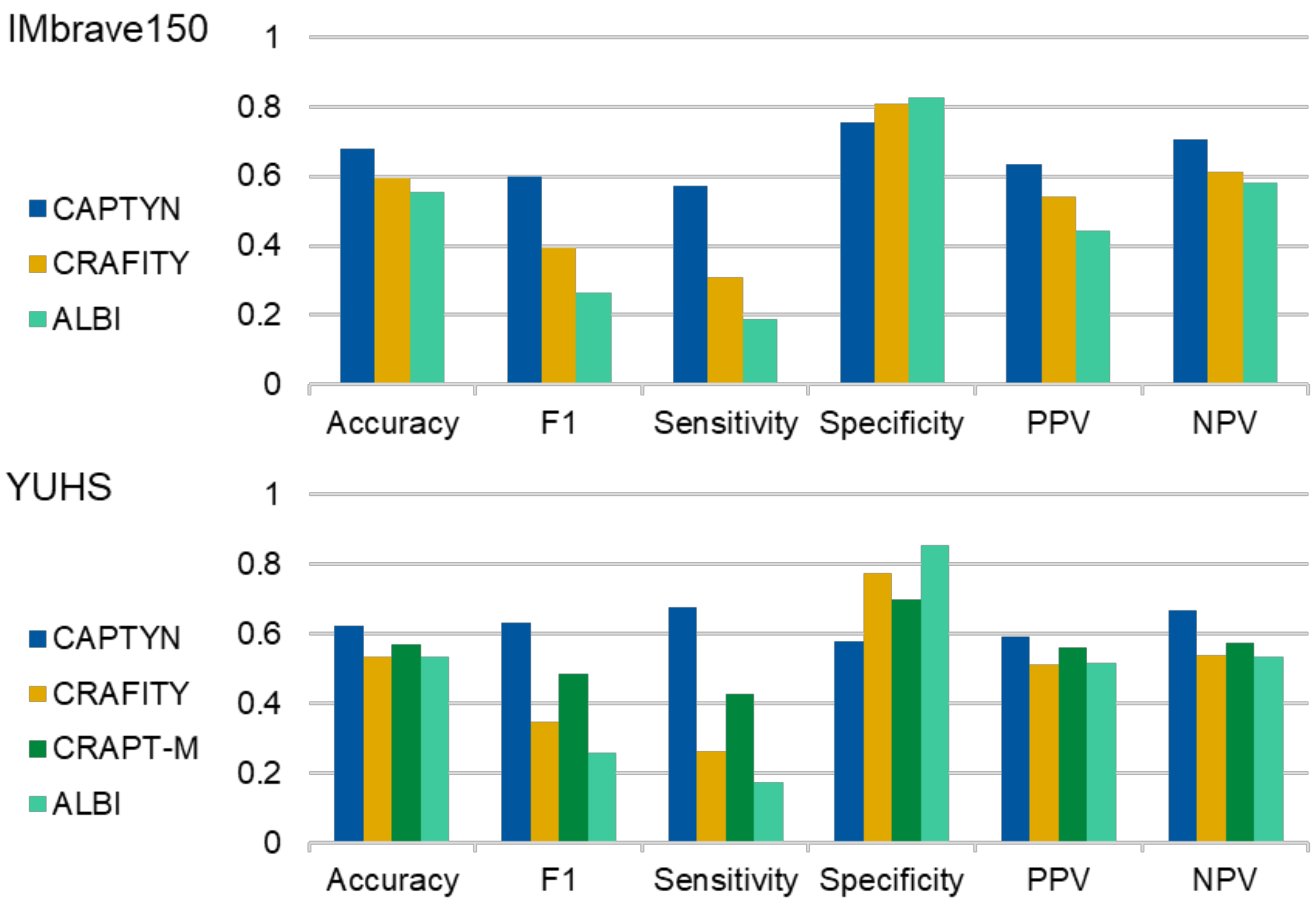

Fig. S11. Binary classification metrics of CAPTYN, CRAFITY, CRAPT-M, and ALBI across both validation cohorts. Classification metrics are shown for the IMbrave150 ($n = 99$) and YUHS ($n = 184$) cohorts. NCB was defined as CAPTYN ≥ 0.486, CRAFITY > 1, CRAPT-M > 12, and ALBI ≥ −2.27; all other cases were classified as CB. The F1 score represents the harmonic mean of precision (PPV) and recall (sensitivity). CB, clinical benefit; NCB, non-clinical benefit; NPV, negative predictive value; PPV, positive predictive value; YUHS, Yonsei University Health System.

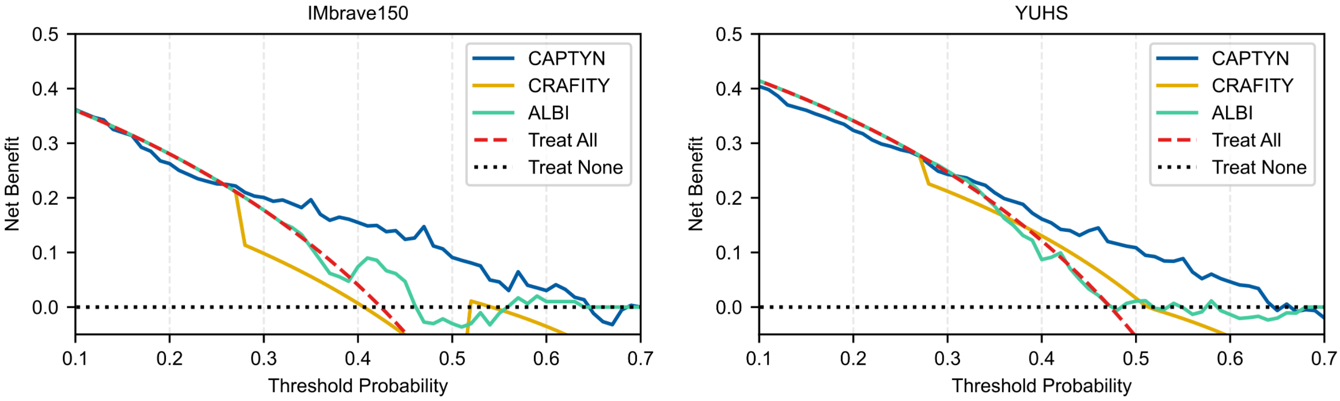

Fig. S12. Decision curve analysis of CAPTYN compared with CRAFITY and ALBI in the IMbrave150 and YUHS cohorts. Decision curves show the net clinical benefit of each model across a range of threshold probabilities for NCB classification. The "treat all" and "treat none" lines represent reference strategies in which all or no patients, respectively, are classified as NCB. CRAPT-M was excluded due to the limited availability of protein induced by vitamin K absence or antagonist-II. NCB, non-clinical benefit; YUHS, Yonsei University Health System.

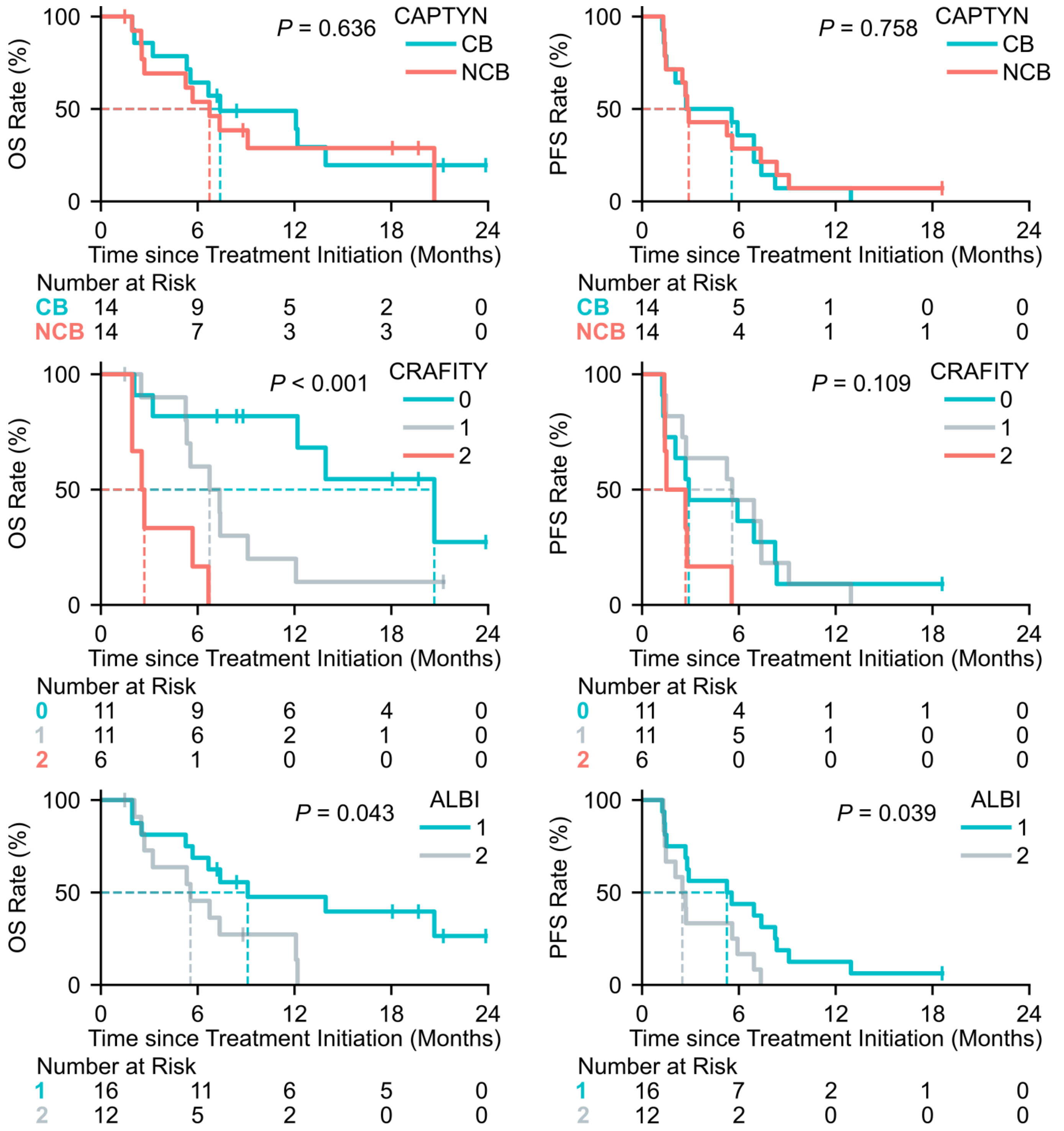

Fig. S13. Kaplan–Meier OS and PFS curves stratified by comparator models in the sorafenib-treated cohort (SOR). Patients in the SOR cohort (n = 28) were stratified by CAPTYN (top), CRAFITY (middle), and ALBI (bottom). CRAFITY scores of 0, 1, and 2 indicate the number of risk factors present (CRP ≥ 1 mg/dL and AFP ≥ 100 ng/mL). ALBI 1 and 2 indicate ALBI grades 1 (score ≤ −2.60) and 2 (score > −2.60 to ≤ −1.39), respectively. Dashed lines indicate median survival. AFP, α-fetoprotein; CB, clinical benefit; CRP, C-reactive protein; NCB, non-clinical benefit; OS, overall survival; PFS, progression-free survival.

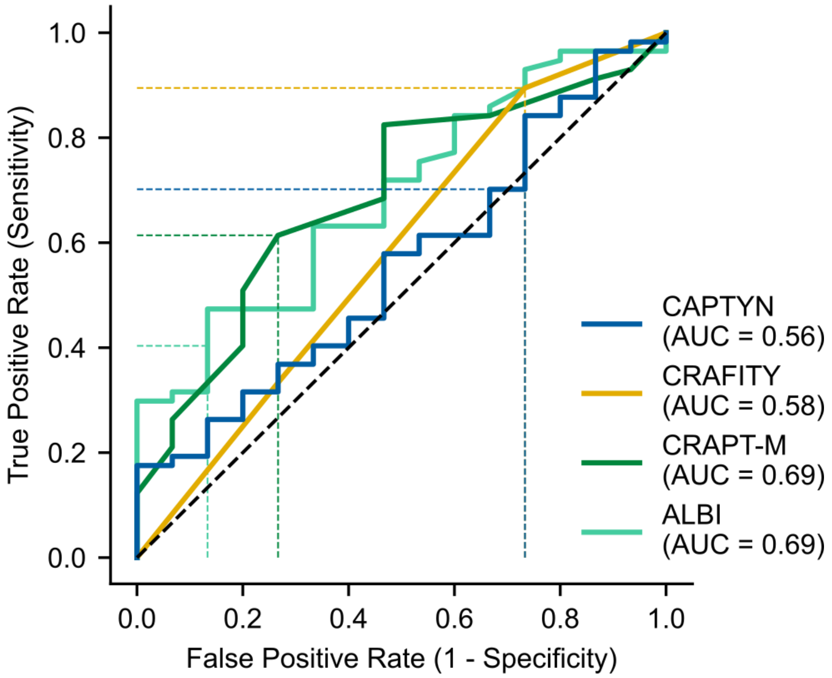

Fig. S14. Receiver operating characteristic curves of CAPTYN, CRAFITY, CRAPT-M, and ALBI in the lenvatinib-treated cohort (LEN). ROC curves and AUC values for prediction of NCB are shown for the LEN cohort (n = 72). In the ROC curves, the diagonal dashed line indicates the line of no discrimination, and the thin dashed lines mark the sensitivity and false positive rate at each model's reference cutoff (CAPTYN ≥ 0.486, CRAFITY > 1, CRAPT-M > 12, and ALBI ≥ −2.27). AUC, area under the curve; NCB, non-clinical benefit; ROC, receiver operating characteristic.

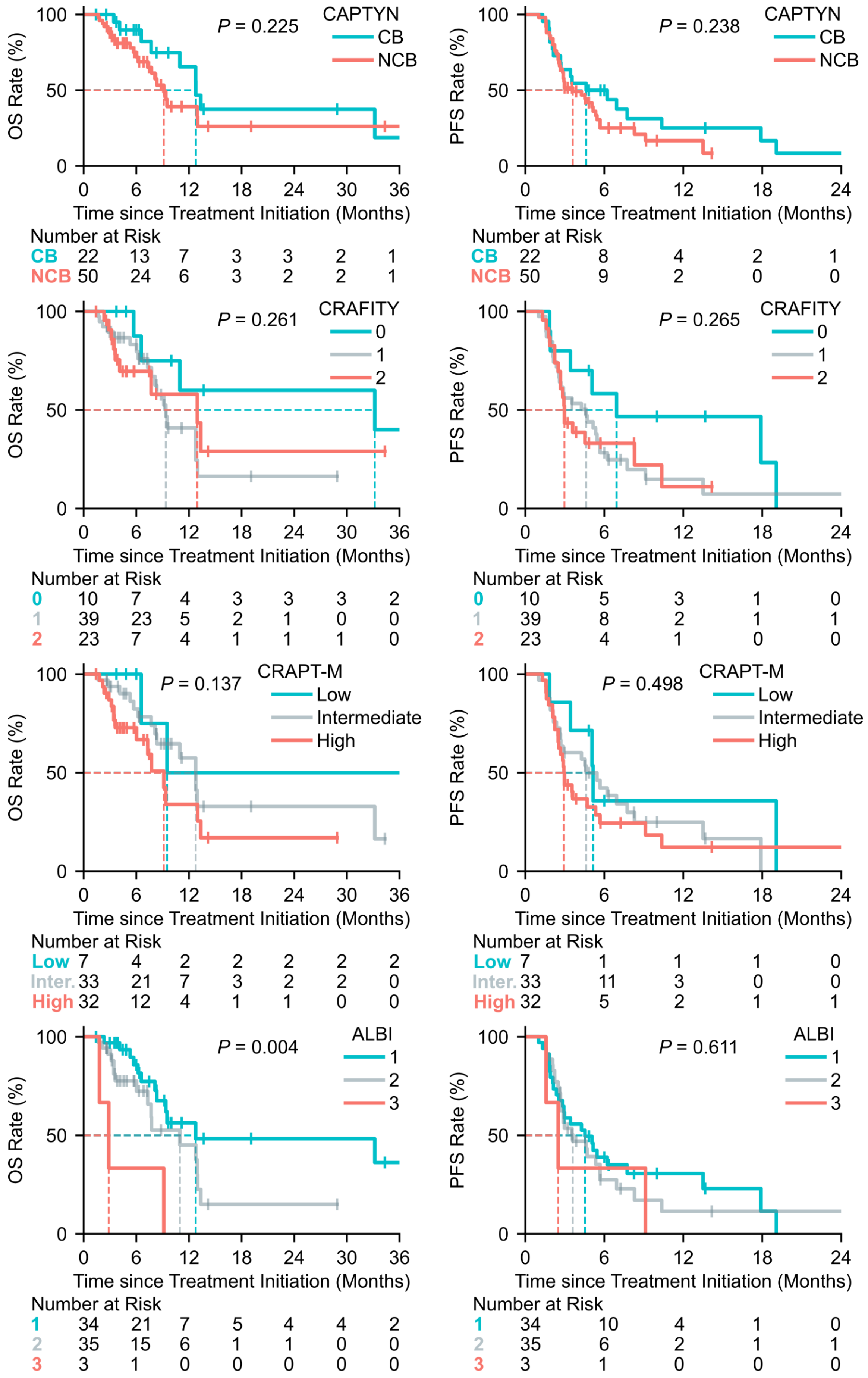

Fig. S15. Kaplan–Meier OS and PFS curves stratified by comparator models in the lenvatinib-treated cohort (LEN). Patients in the LEN cohort (*n* = 72) were stratified by CAPTYN (top), CRAFITY (second

row), CRAPT-M (third row), and ALBI (bottom). CRAFITY scores of 0, 1, and 2 indicate the number of risk factors present (CRP ≥ 1 mg/dL and AFP ≥ 100 ng/mL). CRAPT-M risk groups were defined as low (score ≤ 4), intermediate (5–12), and high (≥ 13). ALBI 1, 2, and 3 indicate ALBI grades 1 (score ≤ −2.60), 2 (score > −2.60 to ≤ −1.39), and 3 (score > −1.39), respectively. Dashed lines indicate median survival. AFP, α-fetoprotein; CB, clinical benefit; CRP, C-reactive protein; Inter., intermediate; NCB, non-clinical benefit; OS, overall survival; PFS, progression-free survival.

Supplementary Tables

Table S1. Baseline characteristics of 737 patients with hepatocellular carcinoma treated with atezolizumab plus bevacizumab or tyrosine kinase inhibitors.

Variable	CHA<br>(*n* = 301)	Vienna<br>(*n* = 53)	IMbrave150<br>(*n* = 99)	YUHS<br>(*n* = 184)	SOR<br>(*n* = 28)	LEN<br>(*n* = 72)
Age (year)	62.00<br>(55.00–69.00)	71.00<br>(63.00–80.00)	64.00<br>(59.00–71.00)	61.00<br>(54.00–70.00)	64.50<br>(56.50–70.25)	61.00<br>(54.00–68.00)
Sex						
Female	45 (15.0%)	17 (32.1%)	21 (21.2%)	29 (15.8%)	4 (14.3%)	8 (11.1%)
Male	256 (85.0%)	36 (67.9%)	78 (78.8%)	155 (84.2%)	24 (85.7%)	64 (88.9%)
Etiology						
Non-viral	83 (27.6%)	33 (62.3%)	35 (35.4%)	52 (28.3%)	10 (35.7%)	10 (13.9%)
Viral	218 (72.4%)	20 (37.7%)	64 (64.6%)	132 (71.7%)	18 (64.3%)	62 (86.1%)
ECOG PS						
0	108 (35.9%)	42 (79.2%)	49 (49.5%)	183 (99.5%)	12 (42.9%)	52 (72.2%)
1	193 (64.1%)	11 (20.8%)	50 (50.5%)	1 (0.5%)	16 (57.1%)	20 (27.8%)
Child–Pugh						
A	238 (79.1%)	36 (67.9%)	99 (100.0%)	177 (96.2%)	28 (100.0%)	67 (93.1%)
B	63 (20.9%)	17 (32.1%)	0 (0.0%)	7 (3.8%)	0 (0.0%)	5 (6.9%)
BCLC stage						
B	56 (18.6%)	21 (39.6%)	10 (10.1%)	23 (12.5%)	3 (10.7%)	5 (6.9%)
C	245 (81.4%)	32 (60.4%)	89 (89.9%)	161 (87.5%)	25 (89.3%)	67 (93.1%)
ALT (IU/L)	27.00<br>(18.00–45.00)	38.00<br>(27.00–55.00)	33.00<br>(22.00–63.00)	25.00<br>(13.00–48.00)	53.00<br>(31.00–77.75)	26.00<br>(18.75–41.50)
Albumin (g/L)	39.00<br>(35.00–42.00)	39.20<br>(36.30–42.20)	39.90<br>(36.20–43.00)	41.00<br>(37.00–44.00)	40.65<br>(38.00–42.70)	39.00<br>(35.00–43.00)
T.Bilirubin (mg/dL)	0.71<br>(0.51–1.09)	0.80<br>(0.56–1.30)	0.80<br>(0.60–1.10)	0.80<br>(0.60–1.20)	0.81<br>(0.52–1.10)	0.90<br>(0.60–1.42)
PT-INR	1.11<br>(1.05–1.18)	1.20<br>(1.10–1.30)	1.10<br>(1.02–1.18)	1.02<br>(0.98–1.09)	1.11<br>(1.07–1.22)	1.10<br>(1.01–1.21)
Neutrophil (10^3/μL)	3.13<br>(2.25–4.40)	3.80<br>(2.63–5.20)	4.01<br>(2.99–5.63)	3.59<br>(2.64–5.00)	4.04<br>(2.96–4.82)	3.76<br>(2.70–5.43)
Lymphocyte (10^3/μL)	1.16<br>(0.82–1.58)	1.20<br>(0.80–1.46)	1.30<br>(0.90–1.87)	1.09<br>(0.76–1.51)	1.30<br>(0.97–1.75)	0.99<br>(0.69–1.44)
Platelet (10^3/μL)	151.00<br>(105.00–214.00)	155.00<br>(115.00–214.00)	186.00<br>(145.50–249.50)	159.00<br>(113.75–225.00)	187.00<br>(133.75–290.75)	149.00<br>(105.00–246.25)
CRP (mg/dL)	0.57<br>(0.18–1.80)	0.57<br>(0.33–1.52)	0.78<br>(0.17–2.12)	0.91<br>(0.35–2.19)	0.68<br>(0.34–2.62)	1.56<br>(0.51–3.77)
MVI						
No	197 (65.4%)	37 (69.8%)	56 (56.6%)	89 (48.4%)	11 (39.3%)	38 (52.8%)
Yes	104 (34.6%)	16 (30.2%)	43 (43.4%)	95 (51.6%)	17 (60.7%)	34 (47.2%)
EHS						
No	106 (35.2%)	31 (58.5%)	32 (32.3%)	74 (40.2%)	11 (39.3%)	19 (26.4%)
Yes	195 (64.8%)	22 (41.5%)	67 (67.7%)	110 (59.8%)	17 (60.7%)	53 (73.6%)
AFP (ng/mL)	111.30<br>(6.80–2,841.50)	43.37<br>(5.78–381.93)	33.71<br>(6.64–3,550.01)	71.85<br>(10.05–3,098.83)	90.67<br>(6.59–1,160.25)	256.95<br>(21.98–8,232.12)
PIVKA-II (mAU/mL)	338.70<br>(40.90–5,501.96)	NA	NA	1,240.00<br>(72.75–10,867.25)	NA	3,170.00<br>(353.00–<br>13,256.25)

Baseline demographic and clinical characteristics are summarized by cohort. Continuous variables are presented as median (interquartile range). Categorical variables are expressed as number (percentage). PIVKA-II was not routinely measured in the Vienna, IMbrave150, and SOR cohorts. AFP, α-fetoprotein; ALT, alanine aminotransferase; BCLC, Barcelona Clinic Liver Cancer; CHA, CHA Bundang Medical Center; CRP, C-reactive protein; ECOG PS, Eastern Cooperative Oncology Group performance status; EHS, extrahepatic spread; LEN, lenvatinib-treated cohort; MVI, macrovascular invasion; NA, not available; PIVKA-II, protein induced by vitamin K absence or antagonist-II; PT-INR, prothrombin time–international normalized ratio; SOR, sorafenib-treated cohort; T.Bilirubin, total bilirubin; YUHS, Yonsei University Health System.

Table S2. Clinical outcomes by treatment cohort.

Treatment	Regimen	Data set	Cohort	Median (months, 95% CI)		CB (*n*, %)	NCB (*n*, %)
				OS	PFS		
Combination immunotherapy	Atezolizumab plus Bevacizumab	Training (*n* = 354)	CHA (*n* = 301)	14.5 (11.8–17.7)	6.4 (4.5–7.7)	156 (51.8) (CR:12, PR:90, SD^{C}:54)	145 (48.2) (SD^{N}:78, PD:67)
			Vienna (*n* = 53)	12.7 (10.9–17.0)	7.0 (3.1–9.2)	27 (50.9) (CR:1, PR:19, SD^{C}:7)	26 (49.1) (SD^{N}:11, PD:15)
		Validation (*n* = 283)	IMbrave150 (*n* = 99)	18.0 (11.6–22.8)	8.3 (5.6–9.8)	57 (57.6) (CR:7, PR:22, SD^{C}:28)	42 (42.4) (SD^{N}:23, PD:19)
			YUHS (*n* = 184)	18.8 (13.9–24.9)	6.0 (4.3–7.6)	97 (52.7) (CR:8, PR:52, SD^{C}:37)	87 (47.3) (SD^{N}:27, PD:60)
Tyrosine kinase inhibitor	Sorafenib	Control (*n* = 100)	SOR (*n* = 28)	7.4 (5.3–12.2)	2.8 (2.1–5.9)	2 (7.1) (CR:0, PR:2)	26 (92.9) (SD:15, PD:11)
	Lenvatinib		LEN (*n* = 72)	11.0 (8.1–13.0)	4.3 (2.9–5.4)	15 (20.8) (CR:0, PR:15)	57 (79.2) (SD:27, PD:30)

CB, clinical benefit; CHA, CHA Bundang Medical Center; CI, confidence interval; CR, complete response; LEN, lenvatinib-treated cohort; NCB, non-clinical benefit; OS, overall survival; PD, progressive disease; PFS, progression-free survival; PR, partial response; SD, stable disease; SD^{C}, SD with PFS $\geq$ 6 months; SD^{N}, SD with PFS < 6 months; SOR, sorafenib-treated cohort; YUHS, Yonsei University Health System.

Table S3. Univariate Cox regression analysis for overall survival and progression-free survival in the training set.

Variables	Overall survival		Progression-free survival	
	HR (95% CI)	*P*	HR (95% CI)	*P*
Age (year)	0.99 (0.98–1.00)	0.162	0.99 (0.98–1.00)	**0.049**
Sex (male *vs.* female)	0.91 (0.65–1.26)	0.568	1.01 (0.73–1.40)	0.941
Etiology (viral *vs.* non-viral)	0.98 (0.74–1.28)	0.863	1.27 (0.97–1.65)	0.078
BCLC stage (C *vs.* B)	1.16 (0.85–1.59)	0.340	1.16 (0.87–1.55)	0.319
Albumin (g/L)	0.92 (0.90–0.95)	**<0.001**	0.98 (0.95–1.00)	**0.037**
Total Bilirubin (mg/dL)	1.43 (1.30–1.58)	**<0.001**	1.32 (1.17–1.50)	**<0.001**
ALT (IU/L)	1.00 (1.00–1.00)	0.512	1.00 (1.00–1.00)	0.619
CRP (mg/dL)	1.13 (1.10–1.17)	**<0.001**	1.14 (1.10–1.18)	**<0.001**
Neutrophil (10^3/µL)	1.15 (1.07–1.24)	**<0.001**	1.09 (1.02–1.17)	**0.010**
Platelet[a] (10^3/µL)	1.002 (1.000–1.003)	**0.021**	1.001 (1.000–1.003)	0.063
Lymphocyte (10^3/µL)	0.64 (0.51–0.80)	**<0.001**	0.81 (0.67–0.98)	**0.034**
$\log_{10}$AFP (ng/mL)	1.27 (1.17–1.38)	**<0.001**	1.18 (1.09–1.28)	**<0.001**
MVI (yes *vs.* no)	1.82 (1.40–2.38)	**<0.001**	1.44 (1.12–1.86)	**0.005**
EHS (yes *vs.* no)	0.92 (0.71–1.19)	0.507	1.10 (0.86–1.42)	0.438

[a]Hazard ratio of platelet was reported to three decimal places because the effect size per 1,000/µL is negligible and rounding to two decimals would mask the observed association. Bold indicates $P < 0.05$. AFP, α-fetoprotein; ALT, alanine aminotransferase; BCLC, Barcelona Clinic Liver Cancer; CI, confidence interval; CRP, C-reactive protein; EHS, extrahepatic spread; HR, hazard ratio; MVI, macrovascular invasion.

Table S4. Performance metrics of the final XGBoost model in the training set.

Metrics	Score (95% CI)	
	**Training set (*n* = 354)**	**Sensitivity analysis (*n* = 379)**
**AUC**	0.93 (0.90–0.96)	0.93 (0.91–0.96)
**Accuracy**	0.88 (0.84–0.91)	0.88 (0.85–0.91)
**F1 score**	0.87 (0.83–0.91)	0.88 (0.85–0.92)
**Sensitivity**	0.87 (0.82–0.92)	0.88 (0.83–0.92)
**Specificity**	0.89 (0.84–0.93)	0.89 (0.84–0.93)
**PPV**	0.88 (0.82–0.92)	0.89 (0.84–0.93)
**NPV**	0.88 (0.83–0.93)	0.87 (0.82–0.92)

Classification metrics are shown for the original training cohort and a worst-case sensitivity analysis in which patients without radiological evaluation (*n* = 25) were assigned to the progressive disease (NCB) group. 95% CIs were calculated using 2,000 bootstrap iterations.

AUC, area under the curve; CI, confidence interval; NCB, non-clinical benefit; NPV, negative predictive value; PPV, positive predictive value.

Table S5. AUC and DeLong's test for CAPTYN versus comparator models in the validation set.

Comparison	IMbrave150		YUHS	
	AUC (95% CI)	*P*	AUC (95% CI)	*P*
CAPTYN	0.70 (0.59–0.81)	ref	0.67 (0.59–0.75)	ref
vs. CRAFITY	0.52 (0.41–0.63)	0.002	0.55 (0.48–0.63)	0.005
vs. CRAPT-M	NA	NA	0.55 (0.47–0.64)	0.006
vs. ALBI	0.56 (0.44–0.68)	0.048	0.53 (0.45–0.61)	0.003

DeLong's test is a nonparametric method used to compare AUCs between correlated ROC curves, providing a *P*-value for differences in discriminative performance between models. CRAPT-M was not evaluated in IMbrave150 because of the unavailability of PIVKA-II.

AUC, area under the curve; CI, confidence interval; NA, not available; PIVKA-II, protein induced by vitamin K absence or antagonist-II; ROC, receiver operating characteristic; YUHS, Yonsei University Health System.

Table S6. Brier scores of CAPTYN compared with CRAFITY and ALBI in the validation set.

Model	Brier score	
	IMbrave150	YUHS
CAPTYN	0.22	0.24
CRAFITY	0.27	0.26
ALBI	0.24	0.25

The Brier score quantifies the accuracy of probabilistic predictions on a scale from 0 to 1, with lower values indicating better performance and 0 representing perfect prediction.

YUHS, Yonsei University Health System.

Table S7. Multivariate Cox regression analysis for overall survival and progression-free survival in the IMbrave150 cohort.

Variables	No. of patients	Overall survival		Progression-free survival	
		HR (95% CI)	*P*	HR (95% CI)	*P*
Age (year)	99	1.00 (0.97–1.03)	0.913	0.98 (0.95–1.01)	0.123
Etiology			0.685		0.162
Non-viral	35	1 (ref)		1 (ref)	
Viral	64	0.87 (0.45–1.70)		0.67 (0.39–1.17)	
ECOG PS			0.153		0.903
0	49	1 (ref)		1 (ref)	
1	50	1.55 (0.85–2.84)		0.97 (0.58–1.61)	
Child–Pugh score			0.241		0.378
5	73	1 (ref)		1 (ref)	
6	26	1.49 (0.77–2.89)		1.29 (0.73–2.25)	
BCLC stage			0.541		0.304
B	10	1 (ref)		1 (ref)	
C	89	0.71 (0.24–2.11)		1.71 (0.62–4.74)	
ALT (IU/L)	99	1.00 (1.00–1.01)	0.156	1.00 (0.99–1.01)	0.721
MVI			0.213		0.741
No	56	1 (ref)		1 (ref)	
Yes	43	1.45 (0.81–2.60)		1.09 (0.66–1.81)	
EHS			0.326		0.487
No	32	1 (ref)		1 (ref)	
Yes	67	0.72 (0.37–1.39)		0.82 (0.46–1.44)	
PT-INR	99	0.68 (0.09–5.15)	0.711	0.79 (0.12–5.32)	0.807
CAPTYN			**< 0.001**		**0.002**
CB	61	1 (ref)		1 (ref)	
NCB	38	2.57 (1.48–4.48)		2.12 (1.31–3.41)	

ALT, alanine aminotransferase; BCLC, Barcelona Clinic Liver Cancer; CB, clinical benefit; CI, confidence interval; ECOG PS, Eastern Cooperative Oncology Group performance status; EHS, extrahepatic spread; HR, hazard ratio; MVI, macrovascular invasion; NCB, non-clinical benefit; PT-INR, prothrombin time–international normalized ratio.

Table S8. AUC and DeLong's test for CAPTYN versus comparator models in the lenvatinib-treated cohort (LEN).

**Comparison**	**AUC (95% CI)**	***P***
CAPTYN	0.56 (0.40–0.72)	ref
vs. CRAFITY	0.58 (0.42–0.75)	0.745
vs. CRAPT-M	0.69 (0.55–0.84)	0.145
vs. ALBI	0.69 (0.55–0.83)	0.192

DeLong's test is a nonparametric method used to compare AUCs between correlated ROC curves, providing a *P*-value for differences in discriminative performance between models. AUC, area under the curve; CI, confidence interval; ROC, receiver operating characteristic.

Table S9. Default settings and hyperparameter grids for the machine-learning models compared during model development.

Model	Default	Hyperparameter
eXtreme Gradient Boosting (XGBoost)	subsample = 1<br>reg_alpha = 0<br>reg_lambda = 1	subsample = 0.5<br>reg_alpha ∈ {0, 1, 10}<br>reg_lambda ∈ {0, 1, 10}
Random forest classifier (RFC)	n_estimators = 100<br>max_depth = 'None'<br>min_samples_leaf = 1	n_estimators = 500<br>max_depth ∈ {'None', 6, 12}<br>min_samples_leaf ∈ {1, 5, 10}
Support vector classifier (SVC)	kernel = 'rbf'<br>class_weight = 'None'<br>C = 1<br>gamma = 'scale'	kernel = 'rbf'<br>class_weight = 'balanced'<br>C ∈ {0.25, 0.5, 1}<br>gamma ∈ {'scale', 0.01, 0.1}
Logistic regression (LR)	solver = 'lbfgs'<br>penalty = 'l2'<br>C = 1<br>l1_ratio = 'None'	solver = 'saga' (for elastic net)<br>penalty ∈ {'l2', 'elasticnet'}<br>C ∈ {0.1, 0.25, 1}<br>l1_ratio ∈ {0.2, 0.5}

reg_alpha denotes L1 regularization. reg_lambda denotes L2 regularization. C denotes the regularization parameter in SVC and LR.

TRIPOD+AI Checklist 1

TRIPOD+AI

Version: 11-January-2024

Section/Topic	Item	Development / evaluation[1]	Checklist item	Reported on page
**TITLE**				
Title	1	D;E	Identify the study as developing or evaluating the performance of a multivariable prediction model, the target population, and the outcome to be predicted	p.1
**ABSTRACT**				
Abstract	2	D;E	See TRIPOD+AI for Abstracts checklist	N/A
**INTRODUCTION**				
Background	3a	D;E	Explain the healthcare context (including whether diagnostic or prognostic) and rationale for developing or evaluating the prediction model, including references to existing models	p.2
	3b	D;E	Describe the target population and the intended purpose of the prediction model in the context of the care pathway, including its intended users (e.g., healthcare professionals, patients, public)	p.2
	3c	D;E	Describe any known health inequalities between sociodemographic groups	p.2
Objectives	4	D;E	Specify the study objectives, including whether the study describes the development or validation of a prediction model (or both)	p.2
**METHODS**				
Data	5a	D;E	Describe the sources of data separately for the development and evaluation datasets (e.g., randomised trial, cohort, routine care or registry data), the rationale for using these data, and representativeness of the data	Sup. Methods
	5b	D;E	Specify the dates of the collected participant data, including start and end of participant accrual; and, if applicable, end of follow-up	Sup. Methods
Participants	6a	D;E	Specify key elements of the study setting (e.g., primary care, secondary care, general population) including the number and location of centres	Sup. Methods
	6b	D;E	Describe the eligibility criteria for study participants	Sup. Methods
	6c	D;E	Give details of any treatments received, and how they were handled during model development or evaluation, if relevant	Sup. Methods
Data preparation	7	D;E	Describe any data pre-processing and quality checking, including whether this was similar across relevant sociodemographic groups	Sup. Methods
Outcome	8a	D;E	Clearly define the outcome that is being predicted and the time horizon, including how and when assessed, the rationale for choosing this outcome, and whether the method of outcome assessment is consistent across sociodemographic groups	Sup. Methods
	8b	D;E	If outcome assessment requires subjective interpretation, describe the qualifications and demographic characteristics of the outcome assessors	Not required
	8c	D;E	Report any actions to blind assessment of the outcome to be predicted	Not required
Predictors	9a	D	Describe the choice of initial predictors (e.g., literature, previous models, all available predictors) and any pre-selection of predictors before model building	Sup. Methods
	9b	D;E	Clearly define all predictors, including how and when they were measured (and any actions to blind assessment of predictors for the outcome and other predictors)	Sup. Methods
	9c	D;E	If predictor measurement requires subjective interpretation, describe the qualifications and demographic characteristics of the predictor assessors	Not required
Sample size	10	D;E	Explain how the study size was arrived at (separately for development and evaluation), and justify that the study size was sufficient to answer the research question. Include details of any sample size calculation	Sup. Methods
Missing data	11	D;E	Describe how missing data were handled. Provide reasons for omitting any data	Sup. Methods
Analytical methods	12a	D	Describe how the data were used (e.g., for development and evaluation of model performance) in the analysis, including whether the data were partitioned, considering any sample size requirements	Sup. Methods
	12b	D	Depending on the type of model, describe how predictors were handled in the analyses (functional form, rescaling, transformation, or any standardisation).	Sup. Methods
	12c	D	Specify the type of model, rationale[2], all model-building steps, including any hyperparameter tuning, and method for internal validation	Sup. Methods
	12d	D;E	Describe if and how any heterogeneity in estimates of model parameter values and model performance was handled and quantified across clusters (e.g., hospitals, countries). See TRIPOD -Cluster for additional considerations[3]	N/A
	12e	D;E	Specify all measures and plots used (and their rationale) to evaluate model performance (e.g., discrimination, calibration, clinical utility) and, if relevant, to compare multiple models	Sup. Methods
	12f	E	Describe any model updating (e.g., recalibration) arising from the model evaluation, either overall or for particular sociodemographic groups or settings	Not required
	12g	E	For model evaluation, describe how the model predictions were calculated (e.g., formula, code, object, application programming interface)	Supplementary file 2
Class imbalance	13	D;E	If class imbalance methods were used, state why and how this was done, and any subsequent methods to recalibrate the model or the model predictions	No_imbalance
Fairness	14	D;E	Describe any approaches that were used to address model fairness and their rationale	Sup. Methods
Model output	15	D	Specify the output of the prediction model (e.g., probabilities, classification). Provide details and rationale for any classification and how the thresholds were identified	Sup. Methods

[1] D=items relevant only to the development of a prediction model; E=items relating solely to the evaluation of a prediction model; D;E=items applicable to both the development and evaluation of a prediction model
[2] Separately for all model building approaches.
[3] TRIPOD-Cluster is a checklist of reporting recommendations for studies developing or validating models that explicitly account for clustering or explore heterogeneity in model performance (eg, at different hospitals or centres). Debray et al, BMJ 2023; 380: e071018 [DOI: 10.1136/bmj-2022-071018]

Training versus evaluation	16	D;E	Identify any differences between the development and evaluation data in healthcare setting, eligibility criteria, outcome, and predictors	Sup. Methods
Ethical approval	17	D;E	Name the institutional research board or ethics committee that approved the study and describe the participant-informed consent or the ethics committee waiver of informed consent	p.5
**OPEN SCIENCE**				
Funding	18a	D;E	Give the source of funding and the role of the funders for the present study	p.6
Conflicts of interest	18b	D;E	Declare any conflicts of interest and financial disclosures for all authors	p.6
Protocol	18c	D;E	Indicate where the study protocol can be accessed or state that a protocol was not prepared	N/A
Registration	18d	D;E	Provide registration information for the study, including register name and registration number, or state that the study was not registered	N/A
Data sharing	18e	D;E	Provide details of the availability of the study data	p.6
Code sharing	18f	D;E	Provide details of the availability of the analytical code [4]	p.6
**PATIENT & PUBLIC INVOLVEMENT**				
Patient & Public Involvement	19	D;E	Provide details of any patient and public involvement during the design, conduct, reporting, interpretation, or dissemination of the study or state no involvement.	No_involve
**RESULTS**				
Participants	20a	D;E	Describe the flow of participants through the study, including the number of participants with and without the outcome and, if applicable, a summary of the follow -up time. A diagram may be helpful.	Figure S1
	20b	D;E	Report the characteristics overall and, where applicable, for each data source or setting, including the key dates, key predictors (including demographics), treatments received, sample size, number of outcome events, follow-up time, and amount of missing data. A table may be helpful. Report any differences across key demographic groups.	STable 1, p.2
	20c	E	For model evaluation, show a comparison with the development data of the distribution of important predictors (demographics, predictors, and outcome).	N/A
Model development	21	D;E	Specify the number of participants and outcome events in each analysis (e.g., for model development, hyperparameter tuning, model evaluation)	Sup. Methods, Table S2
Model specification	22	D	Provide details of the full prediction model (e.g., formula, code, object, application programming interface) to allow predictions in new individuals and to enable third-party evaluation and implementation, including any restrictions to access or re -use (e.g., freely available, proprietary) [5]	Supplementary file 2
Model performance	23a	D;E	Report model performance estimates with confidence intervals, including for any key subgroups (e.g., sociodemographic). Consider plots to aid presentation.	p.3
	23b	D;E	If examined, report results of any heterogeneity in model performance across clusters. See TRIPOD Cluster for additional details [3].	N/A
Model updating	24	E	Report the results from any model updating, including the updated model and subsequent performance	not_required
**DISCUSSION**				
Interpretation	25	D;E	Give an overall interpretation of the main results, including issues of fairness in the context of the objectives and previous studies	p.2,4
Limitations	26	D;E	Discuss any limitations of the study (such as a non -representative sample, sample size, overfitting, missing data) and their effects on any biases, statistical uncertainty, and generalizability	p.4
Usability of the model in the context of current care	27a	D	Describe how poor quality or unavailable input data (e.g., predictor values) should be assessed and handled when implementing the prediction model	Requires six baseline predictors
	27b	D	Specify whether users will be required to interact in the handling of the input data or use of the model, and what level of expertise is required of users	Excel(xlsm)
	27c	D;E	Discuss any next steps for future research, with a specific view to applicability and generalizability of the model	p.4

From: Collins GS, Moons KGM, Dhiman P, et al. *BMJ* 2024;385:e078378. doi:10.1136/bmj-2023-078378

[4] This relates to the analysis code, for example, any data cleaning, feature engineering, model building, evaluation.
[5] This relates to the code to implement the model to get estimates of risk for a new individual.
